# Nanoscale DNA tracing reveals the self-organization mechanism of mitotic chromosomes

**DOI:** 10.1016/j.cell.2025.02.028

**Published:** 2025-03-24

**Authors:** Kai Sandvold Beckwith, Andreas Brunner, Natalia Rosalia Morero, Ralf Jungmann, Jan Ellenberg

**Affiliations:** 1 Cell Biology and Biophysics Unit, European Molecular Biology Laboratory, Heidelberg, Germany; 2 Dept. Biomedical Laboratory Science, Norwegian University of Science and Technology, Trondheim, Norway; 3 Collaboration for Joint PhD Degree between EMBL and Heidelberg University, Faculty of Biosciences, Heidelberg, Germany; 4 Max Planck Institute of Biochemistry, Planegg, Germany; 5 Faculty of Physics and Center for NanoScience, Ludwig-Maximilians-Universität, Munich, Germany; 6 Science for Life Laboratory (SciLifeLab), Solna, Sweden; 7 Karolinska Institutet, KTH Royal Technology College, Stockholm University, Stockholm, Sweden; 8 These authors contributed equally; 9 Lead contact

## Abstract

How genomic DNA is folded during cell division to form the characteristic rod-shaped mitotic chromosomes essential for faithful genome inheritance is a long-standing open question in biology. Here, we use nanoscale DNA tracing in single dividing cells to directly visualize how the 3D fold of genomic DNA changes during mitosis at scales from single loops to entire chromosomes. Our structural analysis reveals a characteristic genome scaling minimum of 6–8 megabases in mitosis. Combined with data-driven modeling and molecular perturbations, we can show that very large and strongly overlapping loops formed by condensins are the fundamental structuring principle of mitotic chromosomes. These loops compact chromosomes locally and globally to the limit set by chromatin self-repulsion. The characteristic length, density, and increasingly overlapping structure of mitotic loops we observe in 3D fully explain how the rod-shaped mitotic chromosome structure emerges by self-organization during cell division.

## In brief

3D visualization of genomic DNA reveals that the characteristic shape of mitotic chromosomes emerges through extrusion of large genomically overlapping DNA loops and their self-repulsion.

## Graphical Abstract

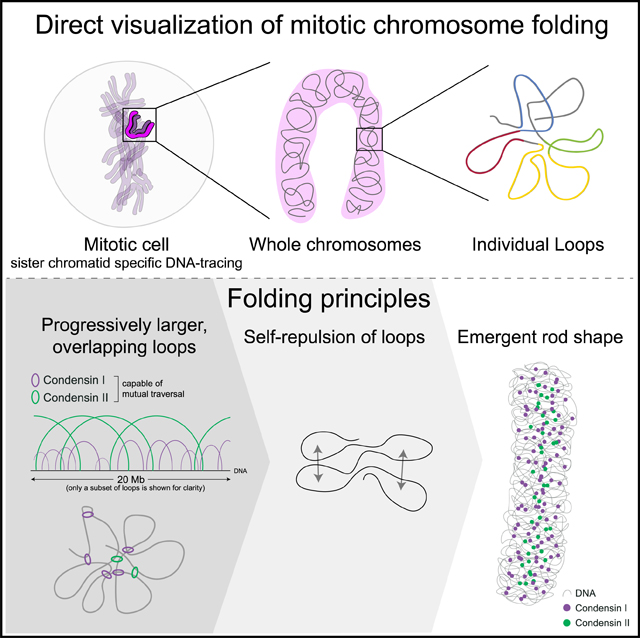

## INTRODUCTION

Upon cell division, chromosomes undergo major shape changes into compact and stiff rod-like entities enabling their segregation into daughter cells.^1–4^ Normal mitotic chromosome structure depends on loop-extruding condensin complexes.^5–8^ Studies using quantitative imaging of condensins in single cells^9^ or biochemical cross-linking of genomic DNA followed by sequencing (High-throughput Chromosome Conformation Capture, or HiC) in synchronized cell populations^10–12^ have hypothesized hierarchical looping models to underlie mitotic chromosome structure. According to these models, the two condensin isoforms should fold chromosomal DNA initially into larger loops (~500 kb) and then subdivide them into smaller loops (~100 kb). Early classical electron microscopy (EM) studies on extracted chromosomes already suggested a loop-like organization of mitotic chromatin,^13^ but the high density of DNA in mitotic chromosomes has precluded direct visualization of its fold in natively fixed cells.^14^ Therefore, the *in situ* internal organization of mitotic chromosomes remains unresolved, and it remains unclear how the higher-order functional properties of mitotic chromosomes for genome inheritance emerge from the molecular-scale structure. Fluorescence-based multiplexed DNA fluorescence *in situ* hybridization (DNA-FISH) (DNA tracing) enables direct visualization of genome organization in single interphase cells.^15,16^ However, these approaches rely on heat and acid denaturation of DNA, potentially disrupting the integrity of nanoscale genome structure.^17,18^ We have previously established a mild, structure-preserving DNA tracing workflow (LoopTrace^17^), based on single-strand resection of DNA,^18–21^ which allows high-fidelity nanoscale tracing of single chromatids *in situ*.^17,22^ Here, we adapt the LoopTrace approach to visualize the organization of mitotic chromosomes from the kilobase to full chromosome scale in single dividing cells, and we use this structural data for polymer simulations, allowing us to propose a self-organization model for mitotic chromosome folding.

## RESULTS

### Multiscale DNA tracing from interphase to metaphase

**Figure 1.**
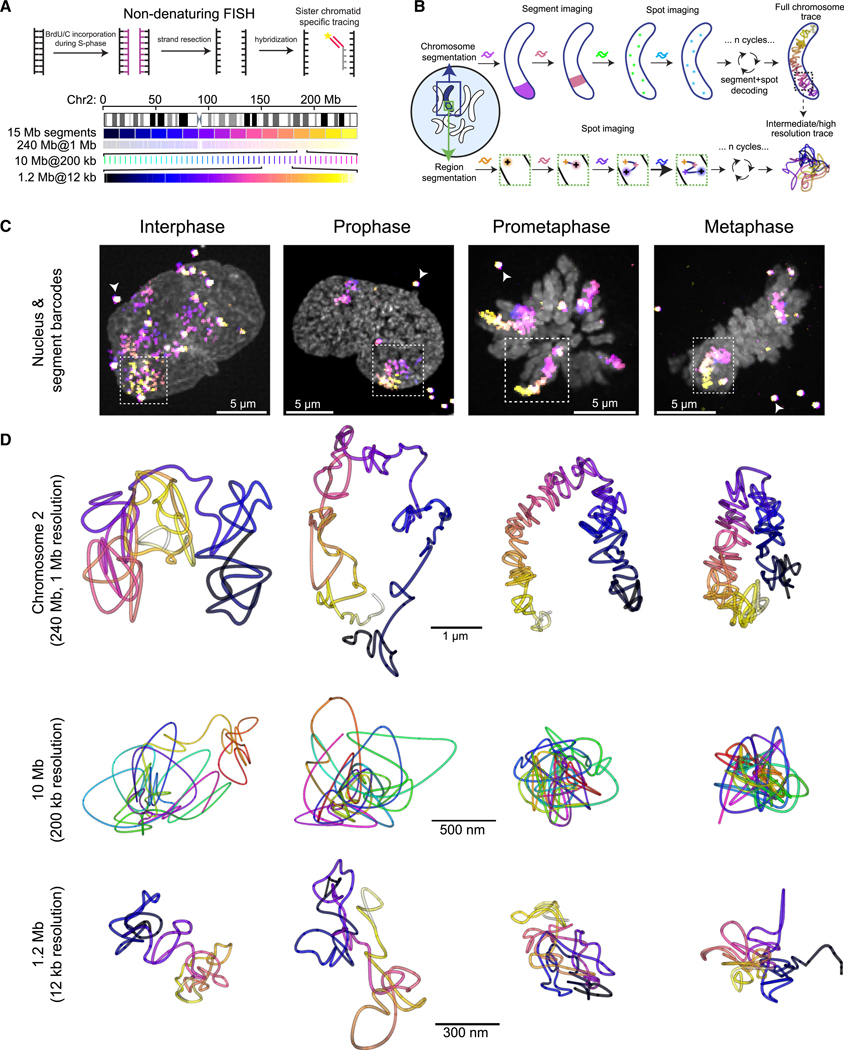
Multiscale DNA tracing from interphase to metaphase (A) Schematic of non-denaturing FISH scheme and multiscale probe libraries targeting chromosome (chr) 2, which was decoded using a combination of 15-Mb segment barcode and 1-Mb resolution spot barcodes. Full chromosome libraries were supplemented with intermediate scale (30-kb spots every 200 kb for 10–12 Mb) and high-resolution (12-kb spots contiguously tiled for 1.2 Mb) libraries targeting the same and different (chr5, chr14, chr18) chromosomes. (B) Schematic of multiscale chromosome trace acquisition. Whole chromosomes were segmented based on the full library signal, while sequential images of 15-Mb segments and 100-kb spots were used to decode and uniquely identify and 3D localize all detected spots. Intermediate and high-resolution libraries were segmented by regional barcodes and traced by sequential spot fitting in 3D. (C) Exemplary maximum-intensity projected micrographs of cell nuclei (DAPI, gray) with full chromosome traces (color-coded sequential 15-Mb segments). Arrowheads indicate fiducial beads used for drift correction. (D) Reconstructed 3D traces (multi-colored) from libraries targeting whole chr2 and intermediate- and high-resolution regions. Data representative of in total 8,879 traces in 1,396 cells in 5 independent experiments. See also Figure S1 and Video S1.

To obtain a direct view of chromosome refolding from interphase to metaphase, we designed FISH probe libraries for tracing entire human chromosomes at 1 Mb genomic resolution (Figures 1A and 1B; Video S1). We chose the 240-Mb sub-metacentric chromosome 2 and the 100-Mb acrocentric chromosome 14 as representative chromosomes. Chromosomes 2 and 14 are both present in two full copies in HeLa Kyoto cells, in addition to a truncated copy of chromosome 2,^23^ which we excluded from further analysis. In addition, we traced three sub-chromosomal 10- to 13-Mb regions at 200-kb resolution, as well as three 1.2-Mb regions at 12-kb resolution. For sub-chromosomal tracing, we used the DNA tracing approach previously established for interphase cells,^15–17^ which is based on centroid fitting of sequentially resolved diffraction-limited DNA-FISH signals (Figure 1B, bottom). For whole chromosomes, we extended this approach by multiplexed tracing of the spots within 15-Mb segments, followed by segment assignment to determine the unique genomic coordinate (Figure 1B, top). After refining our protocols for non-denaturing DNA tracing at the nanoscale^17^ for mitotic cells (Figures S1A and S1B), we could routinely generate multiscale chromosome traces in individual fixed cells during interphase, prophase, prometaphase, and metaphase (Figures 1C and 1D; Table S1; Video S1). Importantly, the non-denaturing FISH protocol only labels one sister chromatid, avoiding issues of signal overlap from paired sisters (Figure 1A).^17–21^ Sequential imaging of the 12-kb-resolved regions achieved a median of 85% trace completeness and better than 25-nm 3D precision, sufficient to reliably resolve individual DNA loops (estimated to be ~100–500 kb^9,11^; Figures S1C and S1D). As expected, interphase chromosomes appeared overall as distinct ellipsoid territories within the cell nucleus,^24^ and DNA looping structures consistent with known Topologically Associated Domains (TADs) were readily visualized at 12-kb resolution (Figures 1C, 1D, S1E, and S1F). Upon entry into mitosis, such genomically reproducibly positioned clusters of small loops were no longer present, and instead variably positioned and increasingly backfolded DNA loops appeared (Figure 1D, prophase to metaphase at 1.2- and 10-Mb scales, and Figures S1E and S1F). At the same time, chromosomes started to individualize into long, thin, and often irregularly bent and variably compacted structures (Figures 1C and 1D). During prometaphase, chromosomes became more contiguously axially compacted, when their arms thickened and straightened, which continued until they reached a compact, rod-like, and often overall V-like shape in metaphase (Figures 1C, 1D, S1G, and S1H).

**Figure 2.**
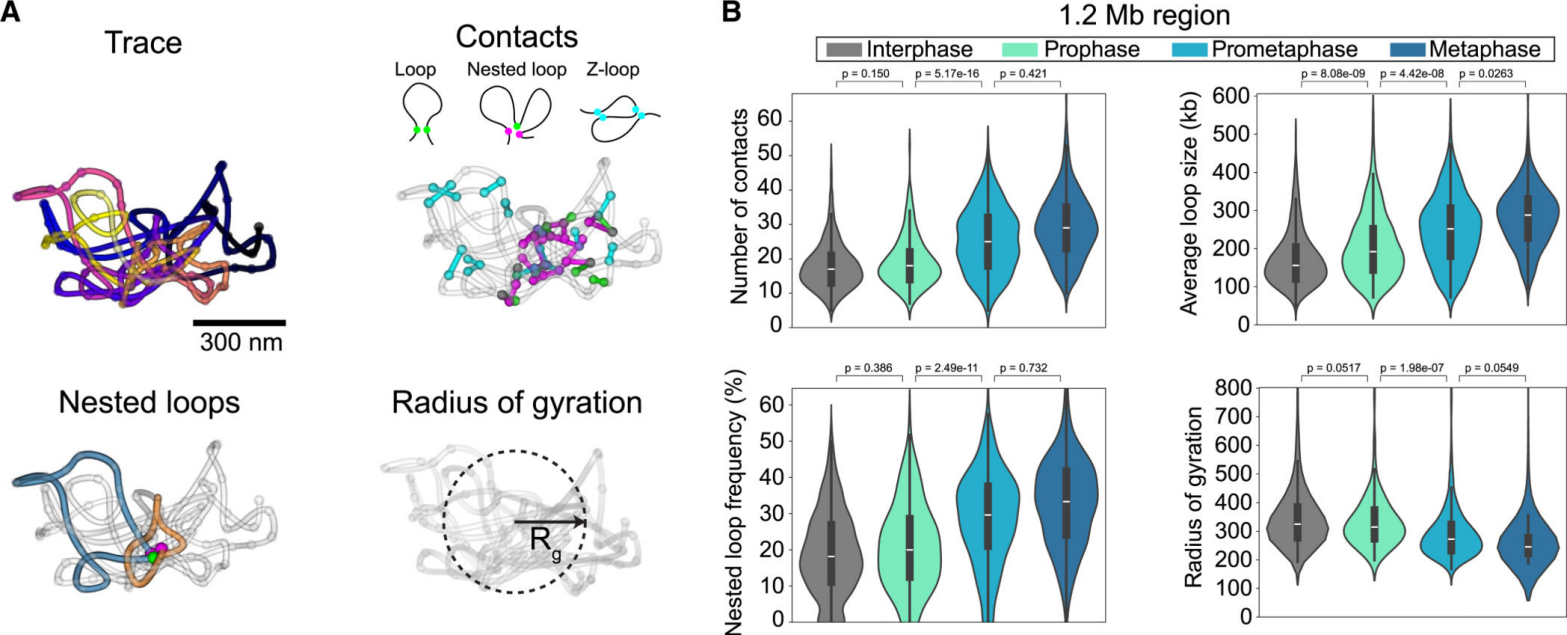
Mitosis-specific chromatin loop signatures (A) Illustration of single-trace metrics. Euclidean distance-based filtering of close contacts (<100 nm) with a genomic distance over 30 kb defines “loops.” Based on their overlap with other loops or loop bases, contacts are classified into base loops, nested loops (when 2 or more base loops coincide to form a second, larger loop), or Z-loops (2 loops partially overlap). Radius of gyration is a measure of overall compaction of the trace. (B) Trace metrics calculated for the high-resolution (12 kb) 1.2-Mb region on chr5:149,500,723–150,699,962. Trace metrics were calculated from traces that were more than 80% complete. Data from *n* = 278 (510), *n* = 22 (44), *n* = 124 (297), and *n* = 152 (398) for interphase, prophase, prometaphase, and metaphase cells (traces) from 3 independent experiments. Median, quartiles, and whiskers are shown in the plots. See also Figure S2.

Having 3D traces from hundreds of mitotically staged single cells allowed us to quantitatively analyze changes in the DNA fold during mitosis, including the abundance, size, and internal nesting of loops, as well as the radius of gyration as a general measure of chromosome compaction (Figures 2A and S2A). Interestingly, mitotic chromosomes exhibited a significant increase in the number and size of loops from prophase onward (Figures 2B and S2B–S2D). This was followed by an increase in nesting of loops with overlapping bases (Figures 2B and S2A, loop measurement illustration, and Figures S2B–S2D) starting in prometaphase when the major wave of condensin I binding occurs.^6,9,25^ Nesting expectedly coincided with a significant decrease of the radius of gyration, indicating that loop-scale compaction occurs mainly in prometaphase (Figures 2B and S2B–S2D). However, the decrease of the radius of gyration began already in prophase, indicating that both loop size increase and loop backfolding and nesting underlie mitotic chromosome compaction. Analyzing these parameters revealed that the structural features that change during mitosis are well sampled at this genomic resolution (Figures S2B–S2D).

The multiscale DNA tracing of mitotic chromosomes also provided an opportunity to examine the question of higher-order regularity of the DNA fold.^3^ We did not observe a highly regular organization at either the whole chromosome, the 10-Mb regions, or at the scale of single loops within 1-Mb subregions, suggesting that loops are stochastically organized relative to the geometric chromosome axis (Figure 1D, metaphase). Consistently, statistical analysis of symmetry parameters relative to the central axis did not show significant helicity at the full chromosome scale (Figure S2E).

### Mitotic chromosome scaling dips at 6–8 Mb

**Figure 3.**
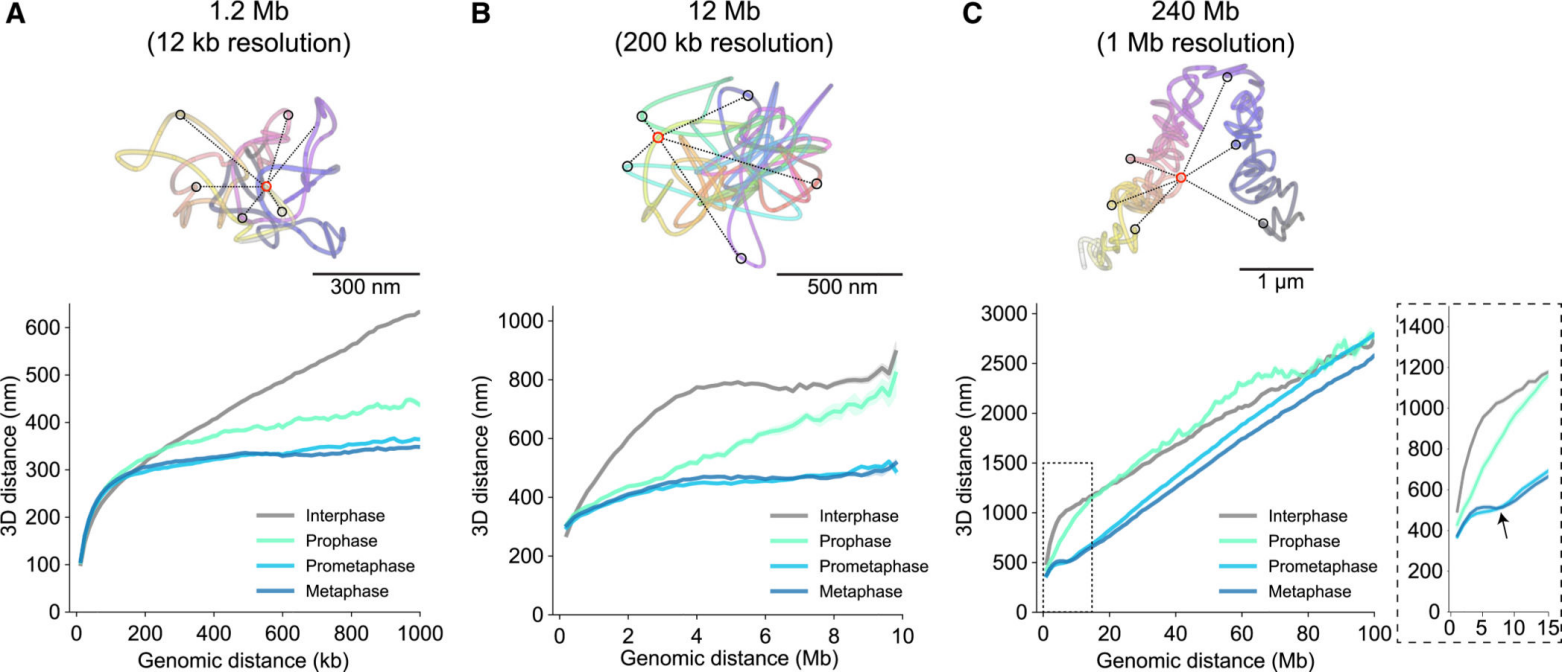
Mitosis-specific genomic distance scaling signatures (A–C) Traces of a high-resolution region (chr5, A), intermediate scale (chr2, B), and whole chr2 (C) and corresponding pairwise distance scaling plots. Exemplary pairwise distances (black) for a single spot (red) within the DNA trace are highlighted. Inset with arrow highlights the characteristic scaling dip at 6–8 Mb. Plots show median ± standard error of the mean. Data from (A) *n* = 400 (824), *n* = 39 (109), *n* = 241 (609), and *n* = 244 (644), from 3 experiments; (B) *n* = 165 (237), *n* = 27 (44), *n* = 175 (325), and *n* = 187 (346), from 2 experiments; and (C) *n* = 331 (417), *n* = 28 (46), *n* = 2 41 (414), and *n* = 212 (357), from 3 experiments; interphase, prophase, prometaphase, and metaphase cells (traces). As the scaling data become very sparse at maximal genomic trace distance, the scaling plots were cropped to 1, 10, and 100 Mb, respectively. See also Figure S3.

If mitotic chromosomes become more compact at each scale, their genomic versus physical 3D distance scaling should also change from interphase to mitosis. To probe distance scaling of chromosomal DNA for additional structural features that might arise during mitosis, we plotted all pairwise Euclidean distances versus the respective pairwise genomic distance for all traces obtained at the three genomic scales, grouped by cell-cycle stage (Figures 3A–3C and S3A–S3C). To quantitatively explore potential power-law scaling in the data, we additionally represented the distance scaling data on logarithmic axes and fit scaling exponents (Figure S3D). Polymers in solution tend to show characteristic power-law scaling behavior of end-to-end distances versus the number of monomers.^26^ A scaling exponent of v = 0.5 indicates an equilibrium globule (Rouse regime), and v = 0.33 indicates a compact fractal globule. Qualitative and quantitative evaluation of our tracing data revealed characteristic scaling behavior of mitotic chromosomes. As expected from the increase in close contacts, mitotic chromosomes showed a significantly higher compaction and strongly reduced scaling exponents at the 200-kb to 1-Mb scale, which started in prophase and were essentially completed in prometaphase (Figures 3A, S3A, S3B, and S3D). Interestingly, this correlated with a slight decompaction of chromatin at short genomic distances of less than 200 kb, consistent with the increased size of mitotic loops (Figures 2B, 3A, S3A, and S3B). Consistently, scaling exponents were similar between all phases below 100 kb at about v = 0.4 (Figure S3D). At the 1- to 10-Mb scales, mitotic chromosomes showed increasing long-range compaction in prophase to prometaphase. Below 3 Mb, prophase, prometaphase, and metaphase scaled similarly with exponents of around 0.15, indicating strong compaction. From 3 to 10 Mb, prophase chromosomes were less constrained (v = 0.41), while prometaphase and metaphase chromosomes were strongly constrained (v = 0.05–0.13), indicating a progressive long-range compaction after prophase (Figures 3B and S3B–S3D).

Not predictable from the loop-scale physical distance metrics, the scaling analysis revealed a higher-order local minimum at 6–8 Mb, indicating increased backfolding of chromatin. This scaling “dip” at large genomic distance appeared in prometaphase and became even more pronounced in metaphase (Figures 3B and S3B–S3D). It was also clearly present at the whole-chromosome scale (Figures 3C, S3B, and S3D), showing that it is a general structural feature and not specific to the three analyzed 10-Mb-scale regions. Consistently, our distance scaling data showed similar scaling features as HiC data from HeLa cells arrested in prometaphase, with the distance scaling dip matching the “second diagonal” described by HiC^11^ (Figure S3E). The whole-chromosome scaling plot furthermore showed that while chromosomes are already significantly more compact in prophase than in interphase at a scale below 10 Mb, they are not yet maximally compacted along their entire length and display a rather irregular scaling behavior above 10 Mb early in mitosis (Figure 3C). We found that axial shortening of chromosomes continued throughout prometaphase and metaphase, with a smooth linear scaling behavior above the 6- to 8-Mb mitotic dip. In line with previous live-cell imaging data,^27^ we found that maximal axial shortening of chromosomes was reached during anaphase (Figures S3F–S3K). Interestingly, the additional axial shortening observed in anaphase occurs alongside a shift to an even larger pairwise distance scaling minimum at around 10 Mb (Figure S3I) and an increase in chromosomal width (Figure S3K).

### Mitotic chromosome structural features depend on condensins

**Figure 4.**
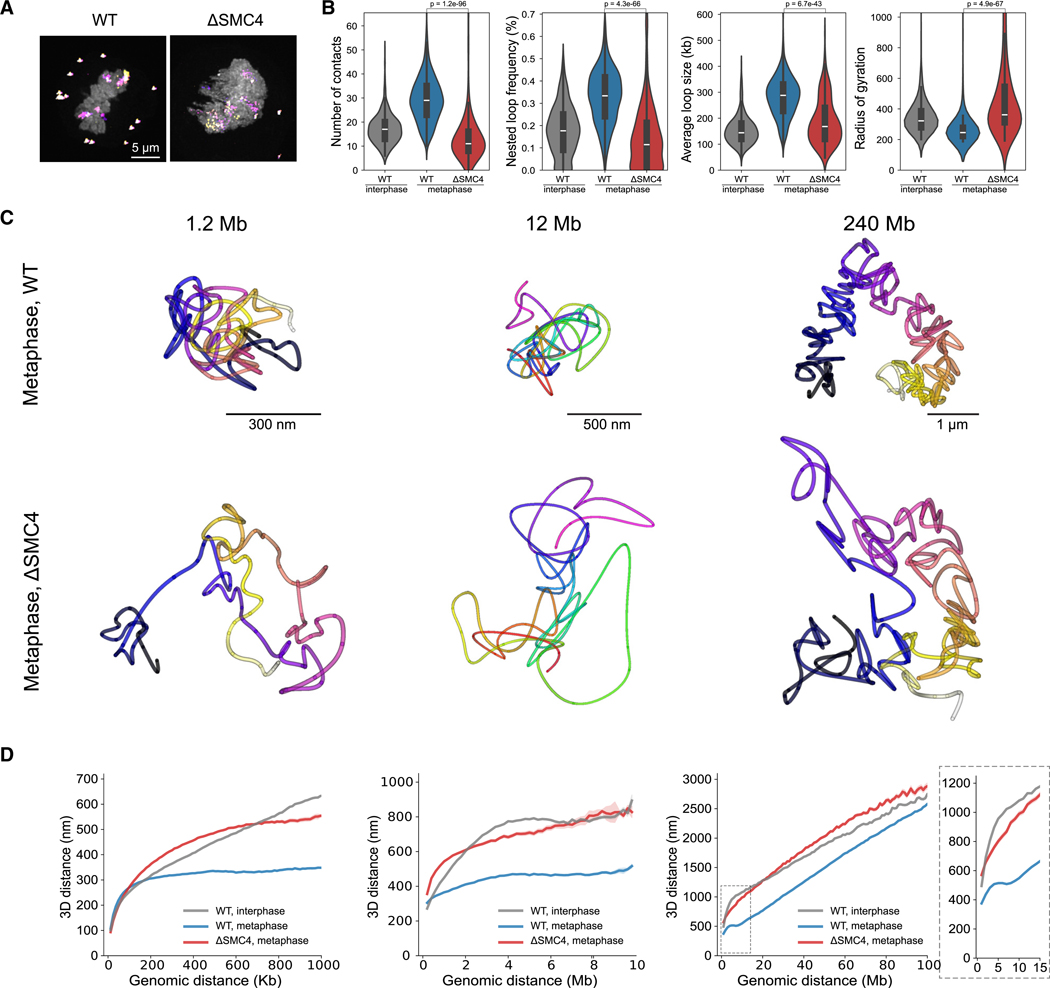
Mitotic chromosome structural features depend on condensins (A) Wild-type (WT) HeLa Kyoto cells and HeLa Kyoto cells with SMC4-mAID-Halo^28^ acutely depleted for 3–4 h with 5-Phenyl-indole-3-acetic acid (5-Ph-IAA) before mitotic entry (ΔSMC4). Exemplary maximum-intensity projected micrograph of metaphase chromatin (DAPI, gray) with full chr2 traces (DNA-FISH, multi-colored). Data representative of 212 WT metaphase traces from 3 independent experiments and 84 metaphase ΔSMC4 traces from 2 independent experiments. (B) Trace metrics from chr5:149,500,723–150,699,962 (1.2 Mb, 12-kb resolution) for WT and ΔSMC4 cells. Data from 152 (398) WT cells (traces), 3 independent experiments, and 153 (265) ΔSMC4 cells (traces), 2 independent experiments. Median, quartiles, and whiskers are shown in the plots. (C) 3D DNA traces for chr5, 1-Mb scale (12-kb resolution); chr2, 10-Mb scale (200-kb resolution); and whole chr2 (1-Mb resolution) from WT and ΔSMC4 cells at metaphase. Data representative of chr5, 1 Mb: 152 (398) WT cells (traces), 3 independent experiments, and 153 (265) ΔSMC4 cells, 2 independent experiments; chr2, 10 Mb: 159 (286) WT cells (traces), 2 independent experiments, and 106 (153) ΔSMC4 cells (traces), 2 independent experiments; chr2 whole: 130 (212) WT cells (traces), 3 independent experiments, and 51 (62) ΔSMC4 cells (traces), one experiment. (D) Distance scaling plots for chr5, 1-Mb scale; chr2, 10-Mb scale; and whole chr2 at metaphase. Data from chr5, 1 Mb: *n* = 400 (824) WT interphase cells (traces) and 244 (644) WT metaphase cells (traces), 3 independent experiments, and 212 (608) ΔSMC4 metaphase cells (traces), 2 independent experiments; chr2, 10 Mb: *n* = 165 (237) WT interphase cells and 187 (346) WT metaphase cells (traces), 3 independent experiments, and 124 (193) ΔSMC4 metaphase cells (traces), 2 independent experiments; chr2, whole: *n* = 331 (417) WT interphase cells (traces) and 212 (357) WT metaphase cells (traces), 3 independent experiments, and 206 (355) ΔSMC4 metaphase cells (traces), 2 independent experiments. As the scaling data become very sparse at maximal genomic trace distance, the scaling plots were cropped to 1, 10, and 100 Mb, respectively. See also Figure S4.

The presence of the directly traced, on average 300-kb-long, mitosis-specific loops that became increasingly nested would be expected to be the result of loop extrusion activity by condensin complexes.^9,11^ To test which structural features of mitotic chromosomes depend on loop extrusion across the sampled scales, we depleted condensins prior to mitosis in HeLa Kyoto cells, with the condensin subunit structural maintanance of chromosomes protein 4 (SMC4) endogenously tagged with an auxin-inducible degron in all alleles.^28^ We then performed multiscale chromatin tracing of condensin-depleted (ΔSMC4) metaphase cells (Figures S4A–S4C) and analyzed the 3D trace parameters as well as their genomic-physical distance scaling (Figures 4A–4D and S4D–S4G). As expected, condensin depletion resulted in a dramatic loss of individual mitotic loops and their nesting and a corresponding decrease in the number of close contacts, demonstrating that mitotic DNA loops and their nesting depend on condensin (Figure 4B). In line with previous reports,^11,29^ SMC4-depleted chromosomes partially retained strong interphase A/B compartments (Figures S4H–S4K), likely as a result of missing condensin-driven loop extrusion. Strikingly, when we examined the distance scaling relationships, the characteristic mitotic dip at 6–8 Mb was completely abolished in ΔSMC4 chromosomes (Figures 4D, S4E, and S4F). In addition, the smooth linear distance scaling behavior above 10 Mb became rather variable after condensin depletion, consistent with the irregular and no longer clearly rod-like shape chromosomes exhibited in the corresponding 3D traces of entire chromosomes (Figure 4C). Consistently, ΔSMC4 chromosomes also failed to reduce their radius of gyration as a measure of general compaction (Figure 4B). Below 100-kb distances, condensin-depleted mitotic chromosomes scaled with an exponent of v = 0.48, close to an equilibrium globule (Figure S4F). At scales of 100 kb–10 Mb, scaling exponents were 0.20–0.27, indicating a compact polymer scaling also in the absence of condensins. Taken together, not only individual mitotic loops and their internal nesting, leading to local compaction, but also the characteristic mitotic 6- to 8-Mb dip and the compaction of entire chromosomes depend on condensins.

### Loop extrusion and self-repulsion correctly predict mitotic chromosome structure

How then could micrometer-scale, rod-shaped mitotic chromosomes arise from molecular-scale architecture of individual DNA loops? Previous theoretical work has shown that in principle, a self-avoiding polymer fiber constrained by cross-links^30^ or loops^31^ could form a rod-like shape through entropic self-repulsion. This is because cross-links/loops backfold the fiber, on the one hand, while the increased density of loops leads to more frequent collisions and a self-repulsion of loops that seeks to maximize the polymer’s available conformational space, on the other hand.^30–33^

While it is clear from our data (Figure 4) and previous work^2^ that the condensin complexes are required for the formation of stiff mitotic chromosome rods, it remained unclear if the generation of loops alone is sufficient to explain our newly determined 3D structure and scaling properties of mitotic chromosomes. To test this mechanistically, we set up a polymer model of mitotic chromosomes constrained by the currently available quantitative experimental data on condensins (see below) to compare it with our DNA tracing data. Building on pioneering work by the Mirny group^34^ and their open-source simulation framework (https://github.com/open2c/polychrom), we simulated a realistic human chromosome of 100 Mb as a chain of 100,000 beads (i.e., 1 kb/bead), ensured volume exclusion through repulsion between monomers, and physically scaled the unconstrained polymer by high-resolution ΔSMC4 traces (Figures 4A, 4D, and S5A). We dynamically simulated condensin-driven loop extrusion by the sequential action of initially 2 stably bound condensin II complexes per megabase for 10 min (prophase to nuclear envelope break down) and then 10 dynamically bound condensin I complexes for an additional 30 min (prometaphase to metaphase; Table S2).^9,22,35^ Where available, we used parameters for condensin abundance and DNA residence time determined *in vivo*, and we used single-molecule *in vitro* measurements for their unidirectional extrusion rate and high traversal frequency.^8,36^ Otherwise, our model was assumption free, especially regarding the structure of the polymer.

**Figure 5.**
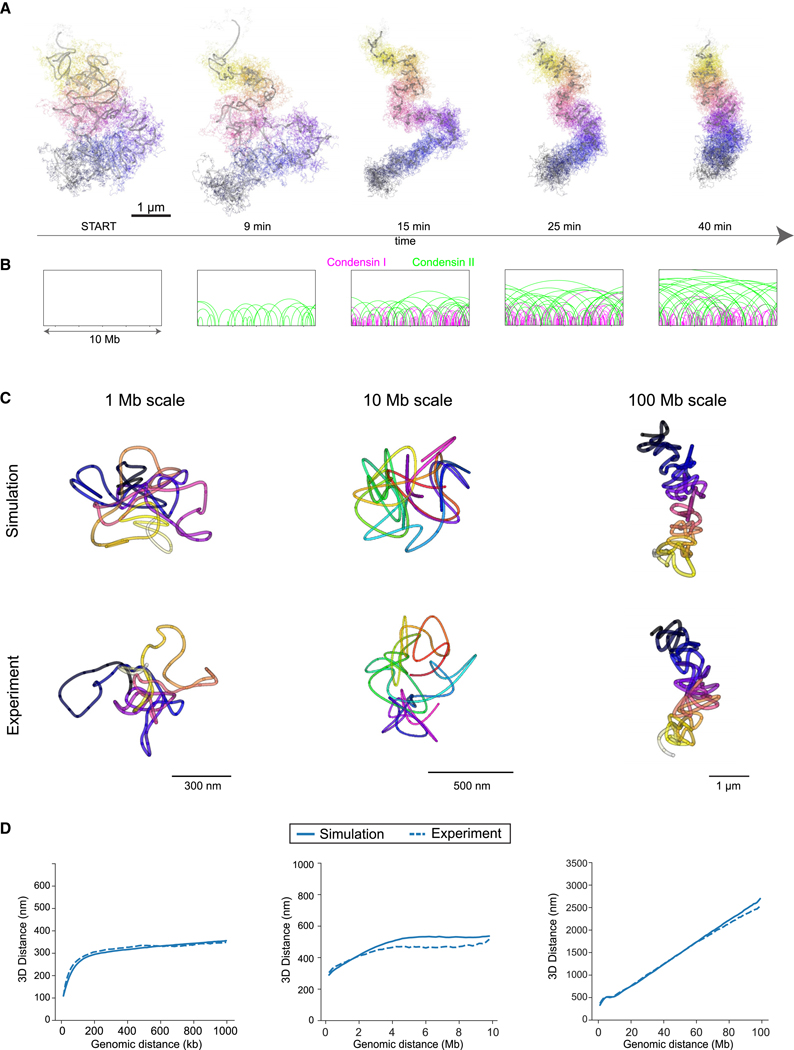
Loop extrusion and self-repulsion correctly predict mitotic chromosome structure (A) Polymer simulation of mitotic progression for a 100-Mb chromosome sampled at the indicated time points, displayed at 2-kb resolution (multi-colored, thin line) and as a 1-Mb rolling average (gray, thick line). Data representative of 20 dynamically simulated chromosomes. (B) Condensin I (magenta) and condensin II (green) loop lengths shown for a 10-Mb stretch of the simulation example shown in (A). Loop height scales with length for clarity. (C) Reconstructed traces from simulated regions from 100-Mb chromosomes at metaphase (40 min) and corresponding experimental data (chr5 1-Mb scale, chr2 10-Mb scale, chr2 100 Mb from q-arm). Simulated traces were sampled as experimental data (12-kb tiled probes, 30-kb probes with 200-kb resolution and 100-kb probes with 1-Mb resolution). Simulated data representative of 20 simulated 100-Mb chromosomes and experimental data representative of chr5, 1 Mb: 152 (398) cells (traces); chr2, 10 Mb: 159 (286) cells (traces); chr2, 100 Mb: 130 (212), 3 independent experiments. (D) Distance scaling plots for simulated metaphase chromosomes and corresponding experimental data. Simulated chromosomes were sampled as in (C), either 10 times (10- and 100-Mb scales) or 60 times (1-Mb scale) with different starting positions in each of 20 100-Mb simulated chromosomes for representative sampling. Experimental data from chr5, 1 Mb: *n* = 244 (644) cell (traces), 3 independent experiments; chr2, 10 Mb: *n* = 187 (346) cell (traces), 2 independent experiments; and chr2q, 100 Mb: *n* = 212 (686) cell (traces), 3 independent experiments. See also Figure S5 and Video S2.

We found that this realistic and data-driven polymer model has an excellent fit to our tracing data across the three experimentally measured scales from prophase to metaphase with an extrusion rate of 4 ± 2 kbps and free traversal of condensins (Figures 5A–5D and S5B–S5E; Video S2). Simulations with lower extrusion rates, stalling, or uniform loop lengths (4 ± 0 kbps extrusion rates) were not able to fully recapitulate the scaling observed in the experimental data (Figure S5B). The simulation shows that condensin-driven loop extrusion is sufficient to generate the experimentally observed mitotic chromosome fold and overall shape. Importantly, the simulation shows that the number of condensin II complexes present in these cells that are stably bound throughout mitosis can extrude overlapping loops of on average 6–8 Mb when they freely traverse one another. The size and overlapping nature of these loops directly explains the distance scaling minimum we observed in prophase/metaphase (Figures 5D and S5B–S5E). By contrast, the number of condensin I complexes present in cells, which are bound only for minutes, leads to nesting inside these large loops, thereby increasing the DNA density and number of loops and thus their self-repulsion. Our simulations furthermore show that loop extrusion and chromatin self-repulsion are sufficient to induce straightening of the mitotic chromosome rod as its length shortens and its width increases (Figure 5A), which is caused by increased self-repulsion of DNA at the concave side of the bent chromosome. Thus, the simulation explains the rod shape and rigidity of chromosomes by a balance of the long-distance compaction forces of large, overlapping loops and the self-repulsion of these loops due to the entropic resistance of the polymer chain to be packed too densely.

### The extrusion-repulsion model predicts realistic condensin localization

If the core mechanism of self-organization is indeed condensin-driven loop extrusion, our polymer model should also be able to predict other structural features of mitotic chromosome architecture. We asked the simulation to output the predicted sub-chromosomal localizations of the two types of condensin complexes in metaphase chromosomes. Without prior assumptions on condensin positioning, the model predicted that condensin II should localize more centrally within the chromosome, while condensin I should localize more peripherally (Figures S5F and S5G). Strikingly, these predictions match previous experimental measurements of condensin isoform localizations within the chromosome by super-resolution microscopy.^9^

We can conclude that self-organization of chromosomal DNA by extrusion of long overlapping loops by condensin II, nested by abundant and dynamic short-range loops by condensin I and their self-repulsion, is sufficient to explain how the experimentally observed structural and higher-order emergent properties of mitotic chromosomes are generated.

### Global compaction is influenced by self-repulsion

**Figure 6.**
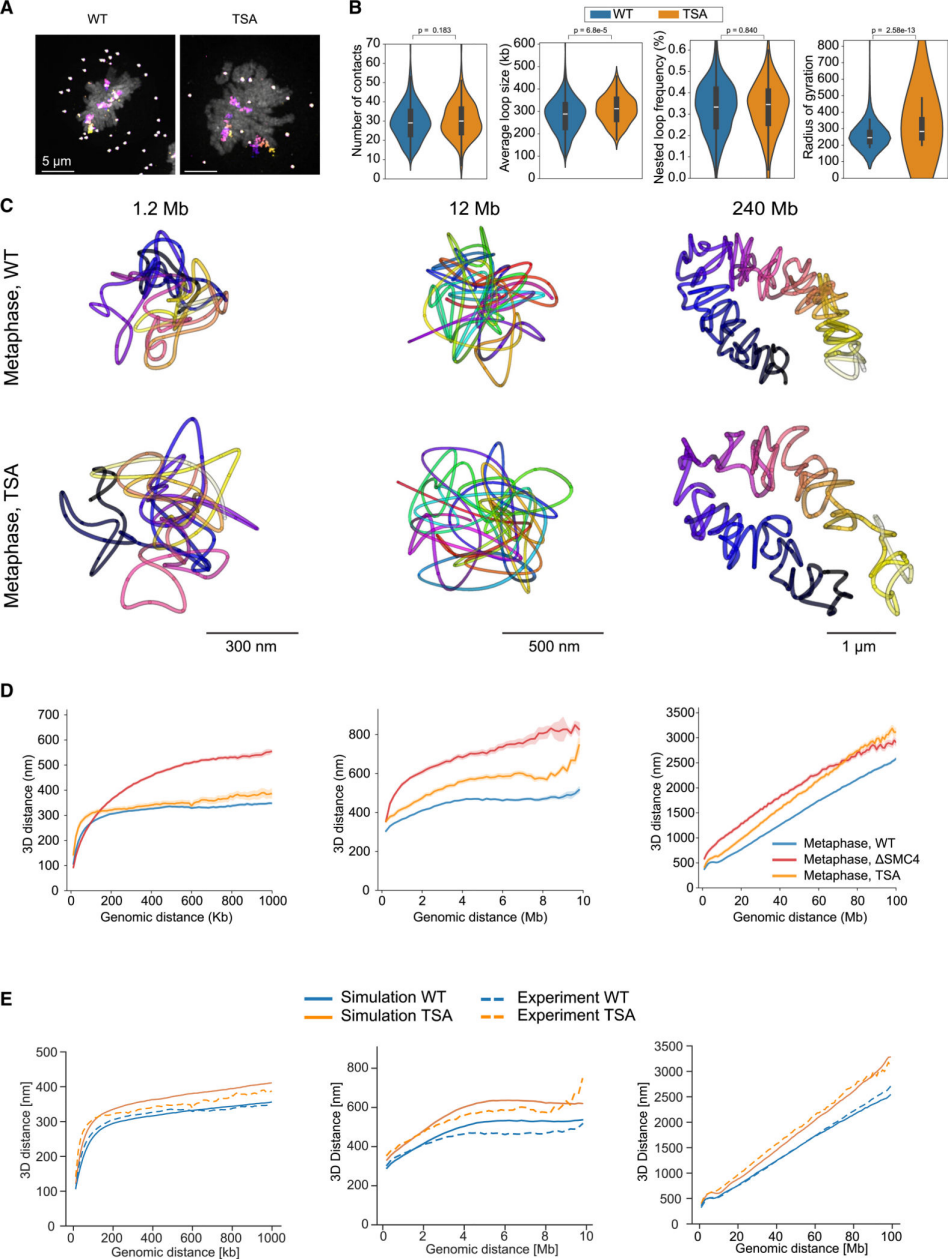
Global compaction is influenced by self-repulsion (A) WT HeLa Kyoto cells and HeLa Kyoto cells treated with TSA before mitotic entry. Exemplary maximum-intensity projected micrograph of metaphase chromatin (DAPI, gray) with full chr2 traces (DNA-FISH, multi-colored). Data representative of 212 WT metaphase cells from 3 independent experiments and 205 prometaphase/metaphase TSA-treated cells from 2 independent experiments. (B) Trace metrics from chr5:149,500,723–150,699,962 (1.2 Mb, 12-kb resolution) for WT and TSA-treated cells. Data from 152 (398) WT cells (traces), 3 independent experiments, and 67 (168) TSA-treated cells (traces), 2 independent experiments. Median, quartiles, and whiskers are shown in the plots. (C) Reconstructed traces for chr5, 1-Mb scale (12-kb resolution); chr2, 10-Mb scale (200-kb resolution); and whole chr2 (1-Mb resolution) from WT and TSA-treated cells at metaphase. Data representative of chr5, 1 Mb: 152 (398) WT cells (traces), 3 independent experiments, and 67 (168) TSA-treated cells, 2 independent experiment; chr 2, 10 Mb: 159 (286) WT cells (traces), 2 independent experiments, and 69 (126) TSA-treated cells (traces), 2 independent experiments; chr2 whole: 130 (212) WT cells (traces), 3 independent experiments, and 19 (31) TSA-treated cells (traces), two independent experiments. (D) Distance scaling plots for chr5, 1-Mb scale; chr2, 10-Mb scale; and whole chr2 at metaphase of TSA-treated cells together with corresponding WT and ΔSMC4 data. Data from chr5, 1 Mb: 71 (198) TSA-treated cells (traces), 2 independent experiments; chr2, 10 Mb: 73 (135) TSA-treated cells (traces), 2 independent experiments; and chr2, whole: 24 (69) TSA-treated cells (traces), 2 independent experiments. See Figures 3 and 4 for details on WT and ΔSMC4 data. As the scaling data become very sparse at maximal genomic trace distance, the scaling plots were cropped to 1, 10, and 100 Mb, respectively. (E) Distance scaling plots from simulated 100-Mb WT and TSA-treated chromosomes, sampled as the corresponding experimental data (see D for details). TSA treatment was simulated by increasing the repulsive potential between monomers. Simulated data from 20 dynamically simulated chromosomes per condition, sampled at metaphase (40 min). See also Figure S6.

In addition to loop extrusion, chromatin modifications have been reported to influence global mitotic chromosome organization.^28^ We therefore asked if we could accurately predict the effect of a change in chromatin modifications, independent of changes to loop extrusion. We thus modeled an experimentally accessible chromatin modification, i.e., the net negative charge of histones, which can be changed with inhibition of histone deacetylases by Trichostatin A (TSA), leading to hyperacetylation of normally positively charged lysines on histone tails.^37^ More negatively charged chromatin should result in a higher self-avoidance, due to electrostatic and/or steric effects.^38,39^ To predict the effect of higher self-avoidance in our model, we increased the monomer repulsion in our simulated polymer and observed a uniform expansion of mitotic chromosomes (Figure 6E). Importantly, the model predicted that this global expansion of chromatin can occur without affecting the condensin-driven loop organization, and thus it predicts that the main structural effect of higher self-repulsion should be to limit the density to which DNA loops can be packed. To validate this prediction experimentally, we performed mitotic chromosome tracing in cells in which histone deacetylase had been inhibited with TSA (Figures 6A and S6A–S6C). TSA-treated cells indeed exhibited unchanged condensin-driven structural features such as loop abundance, size, and nesting, as well as a preserved characteristic scaling dip at 6–8 Mb and similar power-law scaling exponents (Figures 6B–6E and S6D–S6G). By contrast, TSA-treated mitotic chromosomes exhibited a clear, multiscale decompaction of chromatin (Figures 6D and S6E), leading to a 20% expansion of chromatid width and length, consistent with model predictions (Figures 6E, S6H, and S6I). Thus, our model can discriminate between the distinct contributions of loop extrusion and chromatin state in shaping mitotic chromosomes, providing a mechanistic explanation for the observation that TSA treatment leads to global decompaction of mitotic chromosomes.^28^

## DISCUSSION

Here, we present DNA tracing data of native mitotic chromosomes. Combining multiscale 3D DNA tracing in single cells with mechanistic exploration of data-constrained polymer simulations, we have found that the fine-scale structure and the large-scale emergent properties of mitotic chromosomes, including their characteristic rod-like shape, can be explained by a simple self-organization mechanism driven by the counteracting forces of condensin-driven loop extrusion and self-repulsion of DNA.

Extrusion of on average 6- to 8-Mb-long condensin II loops that overlap with one another multiple times in a gapless manner along the whole-chromosomal DNA molecule is a key structuring element of this model. This shortens the axial length of the polymer and enforces stacking of DNA when reaching the self-repulsion limit. Importantly, the progressive formation of long, overlapping loops requires processive loop extrusion as long as condensin is bound to DNA, which has been shown to occur *in vitro*.^8,40^ Yeast condensin is a strictly one-sided loop extruder,^8^ and it was recently proposed that all eukaryotic SMCs extrude loops in a one-sided manner,^41^ although cohesin could effectively extrude symmetrically by directional switching.^41,42^ Both one- and two-sided extrusion modes have been reported for human condensin.^40^ Traversal of condensin motors^36^ is a key assumption to achieve gapless overlaps between loops required for effective mitotic chromosome compaction independent of symmetric or asymmetric modes of loop extrusion.^42^ Thus, although we have used asymmetric extrusion in our model, the model remains compatible with both asymmetric and symmetric loop extrusion by human condensins, as long as they can traverse each other.^8,40,41^ Combined, the processivity while bound and free traversal allow condensin loops to reach the average 6- to 8-Mb size that recapitulates the observed distance scaling minimum. The redundancy in loop overlaps also explains the robustness of mitotic chromosome shape, despite varying condensin abundances observed between single mitotic cells.^9^ Furthermore, redundantly overlapping loops provide an explanation for the mechanical resilience of mitotic chromosomes without requiring a contiguous condensin-based axis.^2,4^

We found that an extrusion rate of 4 ± 2 kbps in our model could best recapitulate the long and overlapping condensin II loops within the ~40-min duration from prophase to metaphase in order to match the experimentally measured mitotic compaction and scaling behavior of whole chromosomes. This estimate is similar to rates estimated from HiC data from chicken DT40 cells (4 kbps in Gibcus et al.^11^ and 1–3 kbps in Samejima et al.^12^) and slightly higher than previous estimates based on *in vitro* single-molecule studies of human, yeast, and Xenopus condensins (0.2–2.4 kbps/s^8,40,43^). It is worth bearing in mind, however, that *in vitro* extrusion rates may differ from *in vivo* rates because of a different physiological state of chromatin, such as differences in DNA tension.^43,44^ We anticipate that future studies on the biophysical mechanism of condensin loop extrusion under more physiological conditions will help to obtain better estimates of the *in vivo* extrusion rate.

Our results are consistent with earlier contact-frequency analyses of mitotic cell populations by HiC^10,11^, including loss of loops, TADs, and compartments during mitosis and the emergence of similar scaling features. In addition, we provide a fully self-consistent model for how the action of the two condensin isoforms explains major features of both nanoscale and macroscale organization of mitotic chromatin. Compared with previous models based on HiC data,^11,12^ the key differences include that our model does not impose an external constraint to global chromosome shape or helicity and allows free traversal of condensins.

Our model predicts that through the generation of increasingly long and overlapping loops from prophase to metaphase (Figures 2B, 3A–3C, and 4A–4D), condensin II effectively decreases the length of chromatids while increasing their width. In contrast, the more abundant and dynamically bound condensin I complexes^9,35^ lead to smaller, short-lived loops nested inside these larger loops, which induce a lateral compaction and increase the self-repulsion of the polymer. This self-repulsion in turn constrains the maximal longitudinal compaction condensin II can achieve and leads to the formation of the mitotic chromosomal rod shape. Our model thus provides a mechanistic explanation for previous observations of global changes to chromosome shape resulting from isoform-specific condensin depletion.^5,6,45^

Previously, a higher-order regular folding of mitotic chromosomes into a helix or a perverted helix has been proposed.^11–13,46–48^ While our direct 3D tracing data do not provide support for a regular helix, our polymer simulation can be used to explore under what conditions it could emerge. Key would be that condensin II motors would extrude loops of highly reproducible length, which would result in regular undulations in pairwise distance scaling from an initial minimum, a characteristic of a regular helix (see Figure S5D). However, this does not match the experimental data we obtained in cultured human cells. Rather, our direct 3D tracing suggests a wide distribution of condensin II loop lengths, resulting in the characteristic distance scaling dip at the average loop size of 6–8 Mb, without the formation of a regular, handed helix in individual chromosomes (see Figure S2E).

In conclusion, by providing the first direct visualization of the internal fold of mitotic chromosomes, we have been able to propose a realistic model of the self-organization principles of DNA that allow faithful genome inheritance. This model is constrained by quantitative experimental data and free of assumptions regarding chromosome structure. The counteracting forces of gathering the linear DNA molecule into long, overlapping loops and limiting their packing by self-repulsion are sufficient to explain the experimentally determined structures by polymer self-organization.

### Limitations of the study

Previous chromatin tracing efforts relied on denaturing FISH protocols, during which the double-stranded DNA is opened up using acid and heat treatment.^15,16^ This method leads to pervasive macro- and microstructural rearrangements of DNA, which likely prevents faithful high-resolution tracing of chromatin.^17,18^ Using enzymatic non-denaturing FISH in combination with chromatin tracing (LoopTrace), we previously reported a high structural preservation of nuclear DNA, thus enabling the imaging of nanoscale features of chromatin, such as DNA loops.^17^ While this method significantly improved the preservation of entire nuclei as well as replication domains, we cannot exclude small-scale structure perturbations due to the lack of knowledge of the ground truth. However, our chromatin tracing data are able to reproduce DNA loop structures known through orthogonal HiC data with high trace fidelity and revealed the existence of DNA loops in single cells^17^ (Figures S1E and S1F), suggesting that the remaining structural perturbation is limited.

This study uses the HeLa Kyoto cell line, a widely used model system for the interrogation of human mitotic and interphase chromosome architecture. While many structural rearrangements and mutations are documented for the HeLa cell model, we do not expect an impact on the action of the condensin loop extruders as they act mostly DNA-sequence agnostic.^8,49,50^ The extent to which our findings hold true in other human cell types and whether they are transferable to animal cells remains to be tested. Nevertheless, the strong conservation of condensin isoforms across the animal kingdom^51^ and the conserved isoform-specific impact on chromosomes, which we can now explain mechanistically,^5,6,11,45^ are strong indications that extrusion of DNA loops and their self-repulsion are essential for mitotic chromosome rod formation.

## STAR★METHODS

### RESOURCE AVAILABILITY

#### Lead contact

Further information and requests for resources and reagents should be directed to and will be fulfilled by the lead contact, Jan Ellenberg (jan.ellenberg@embl.de).

#### Materials availability

This study did not generate new reagents.

#### Data and code availability

All chromatin tracing data generated in this study are available at Figshare: https://doi.org/10.6084/m9.figshare.27003022. Custom Python code to run loop extrusion simulations and prepare polychrom polymer simulations and additional analysis and visualization code examples are available at Figshare: https://doi.org/10.6084/m9.figshare.27003022. Code used to process sequential images and extract DNA traces is available at https://git.embl.de/grp-ellenberg/looptrace. Code used to control the automated fluidics and microscopy acquisition setup is available at https://git.embl.de/grp-ellenberg/tracebot.

### EXPERIMENTAL MODEL AND STUDY PARTICIPANT DETAILS

#### Cell strains and culture

HeLa Kyoto cells (female, HK WT: S. Narumiya (Kyoto University, Kyoto, Japan), RRID: CVCL_1922; HK SMC4-mAID-Halo, AAVS1-OsTir1 F74G-SNAP^28^) were grown in cell culture dishes (Falcon) in high-glucose DMEM (41965–062, Thermo Fisher Scientific) containing 10% FCS (10270–106, Lot. 42F2388K, Thermo Fisher Scientific), 100 U/ml penicillin-streptomycin (15140–122, Thermo Fisher Scientific) and 1 mM sodium pyruvate (11360–039, Thermo Fisher Scientific). Cells were cultured in a humidified incubator at 37°C and 5% CO_2_, unless otherwise stated. Cells were grown to a confluency of 70–90% and passaged every 2–3 days via trypsinization with 0.05% Trypsin-EDTA (25300–054, Thermo Fisher Scientific). Cell lines were regularly checked for Mycoplasma contamination and confirmed negative. To enable non-denaturing FISH via single strand-resection, cells were incubated with 40 μM BrdU/BrdC (ratio 3:1, BrdU: B5002, Sigma-Aldrich, BrdC: sc-284555, Santa Cruz Biotech) for 18–24 h prior to fixation (see cell synchronization).

### METHOD DETAILS

#### Mitotic shake-off

For the enrichment of mitotic cells (e.g. prometa-/metaphase) in a microscopy slide, a mitotic shake-off procedure was used. Asynchronous HK cells were seeded into T-175 flasks (175 cm^2^ surface area) and grown for 18–24 h in the presence of 40 μM BrdU/BrdC to achieve a final confluency of 70–90%. Prior to mitotic shake-off, the medium was aspirated and replaced with 12 mL fresh DMEM. Mitotic cells were detached from the cell culture dish by knocking the culture dish 5x vertically onto a table covered with 5 paper tissues. Cells were collected in a 15 mL Falcon tube and centrifuged at 90xg for 3 min. Cells were resuspended in 100–150 μL fresh medium to a concentration of 2–3.5 ×10^6^ cells/mL. 35 μL of enriched mitotic cells were seeded into each channel of an Ibidi glass-bottom μ-slide (80607, Ibidi), pre-coated with poly-L-lysine (P8920, Sigma-Aldrich) for better attachment of mitotic cells. After 2 min incubation at 37°C, cells were washed once with 1X PBS and fixed using 2.4% paraformaldehyde (PFA) (15710, EMS) in PBS for 15 min at room temperature (RT). PFA was quenched using 100 mM NH_4_Cl in PBS for 10 min.

#### Synchronization with targeted perturbation

For enrichment of cells in prophase and for pre-mitotic degradation of Condensins or inhibition of histone deacetylases with trichostatin A (TSA, Sigma-Aldrich, T8552), cells were pre-synchronized using mitotic shake-off and synchronized in the next mitosis using the Cdk1 inhibitor RO-3306 (Sigma-Aldrich, SML0569).

One day prior to mitotic shake-off, cells were seeded into a T-175 flask in the absence of BrdU/BrdC to reach a confluency of 70–90%. Mitotic shake-off was performed as described above, resuspended cells were diluted to 0.6–0.9 ×10^6^ cells/mL and 35 μL were seeded into a channel of an Ibidi μ-Slide. Cells were allowed to adhere for 20–30 min at 37°C. Subsequently, medium was exchanged to 120 μL DMEM supplemented with 40 μM BrdU/BrdC and cells were incubated for 15 h at 37°C, 5% CO_2_. Cells were synchronized at the G2/M transition using 10 μM RO-3306 for 7–8 h in the presence of BrdU/BrdC. During this time, perturbations were performed. To degrade Condensins, SMC4-mAID-Halo cells^28^ were treated for 3 h with 1 μM 5-Ph-IAA (30–003, BioAcademia). Inhibition of histone deacetylases was performed in HK WT cells using 5 μM TSA for 5 h. Cells were released into mitosis by washing out RO-3306 3x with fresh DMEM. Unperturbed HK WT cells were released for 12 min until fixation using PFA to enrich cells in prophase. ΔSMC4 cells were allowed to enter mitosis for 30 min in the presence of 1 μM 5-Ph-IAA until fixation to reach a metaphase-like state. TSA-treated cells were allowed to enter mitosis for 30–40 min in the presence of 5 μM TSA until fixation. TSA-treated cells, however, failed to align in a metaphase plate as described recently.^28^ Cells were permeabilized for 20 min with 0.25% Triton X-100 (T8787, Sigma-Aldrich) in 1X PBS at RT (T8787, Sigma-Aldrich) for immunofluorescence and non-denaturing FISH. As fiducials for image registration, samples were incubated with 0.1 μm Tetraspec beads (T7279, Thermo Fisher) diluted 1:100 in 1X PBS for 10 min at RT.

#### Library design and amplification

We generated FISH-probe libraries for the entire length of chromosome 2 and chromosome 14 (1 Mb resolution, spot size: 100 kb), the intermediate chromosomal length scale of 11–13 Mb (Chr2: 185,000,034–194,829,658; Chr14: 45,200,003–56,430,006; Chr18: 50,000,077–62,829,855; 200 kb genomic resolution, spot-size: 30 kb) and the megabase scale (Chr2: 191,110,000–192,309,980; Chr5: 149,500,723–150,699,962; Chr14: 50,923,534–52,104,383; 12 kb genomic resolution and spot size). OligoFISH probes were selected and filtered using oligoMiner.^53^ Genome complementary sequences were 36–42 nt long with a melting temperature of 42–46°C (adjusted for 50% formamide, 2X SSC), and specificity filtered by oligoMiner linear discriminant analysis 42°C model with a probability threshold of 0.9, followed by a jellyfish 18 bp k-mer filter with k=10 (k=20 for 1 Mb regions). 20 bp sequences for PCR-amplification were appended to all probes and used during FISH to identify each full chromosome, 10 Mb or 1 Mb region. For probes in 1 Mb and 10 Mb regions, duplicate 12-bp spot-specific barcodes were appended for probes targeting subsequent 12 kb (densely tiled) or 30 kb (spaced by 200 kb) windows, while for full chromosomes each probe had a single 15 Mb segment-specific barcode and duplicate spot-specific barcode targeting each 100 kb (chromosome 2) or 30 kb (chromosome 14) region spaced by 1 Mb. For 1 Mb regions, all filtered probes were used, giving an average of 150 probes per 12 kb spot. Probes for 10 Mb regions and full chromosomes were randomly sampled so that each position had at most 200 and 170 probes, respectively. All probe sequences are listed in Table S3. Libraries were ordered as oligo pools (Genscript). 12-bp barcode sequences were designed as described before.^17^ Imager probes targeting 12-bp barcodes were ordered with a 5’-azide (Metabion GmbH) and labelled with Cy3B-alkyne (ABD-944, AAT Bioquest) or Atto643-alkyne (AD 643–141, Attotec) using click chemistry (ClickTech Oligo Link Kit, BCK-OL-L, Baseclick GmbH) according to the manufacturer’s instructions. Oligo pools were amplified by PCR, followed by *in vitro* transcription and subsequent reverse transcription, as described before.^17,54^

#### Immunofluorescence

Immunofluorescence of Condensin’s SMC2 subunit was performed to visualize chromatid axes relative to the DAPI/tracing signal. After permeabilization and addition of fiducial beads, cells were incubated with blocking buffer (2% BSA, 0.05% Triton X-100 in 1X PBS) for 30 min at RT. Following blocking, cells were incubated with the primary antibody against SMC2 (ab10412, Abcam, 1:1000) in blocking buffer overnight at 4°C or for 2 h at RT. Subsequently, cells were washed 3x for 5 min in blocking buffer and then incubated with goat anti-rabbit AF488 secondary antibody (A-11034, Thermo Fisher, 1:1000) for 1 h at RT in blocking buffer. Cells were washed 3x for 5 min in 1X PBS.

#### Non-denaturing FISH

Non-denaturing FISH (Resolution After Single-strand Exonuclease Resection [RASER]-FISH^19^) was performed as described recently.^17,22^ Permeabilized cells with fiducial beads (and optionally immunofluorescence staining) were incubated for 15 min with 0.5 ng/μL DAPI (D9542, Sigma-Aldrich) and subsequently washed 2x with 1X PBS to sensitize BrdU/C-labeled cells for UV treatment. UV treatment of cells was performed by exposing the Ibidi μ-slide without a lid to 254 nm UV light for 15 min (Stratalinker 2400 fitted with 15W 254 nm bulbs, G15T8) to induce single strand nicks. Nicked DNA was digested using Exonuclease III (M0206, NEB) at a final concentration of 1 U/μL in NEB buffer 1 for 15 min at 37°C in a humidified chamber. Cells were washed 3x with 1X PBS. Cells were post-fixed with 5 mM Bis(NHS)PEG5 (803537, Sigma-Aldrich) in 1X PBS for 30 min at RT to preserve sample fixation during primary probe hybridization. The sample was equilibrated with 1X hybridization buffer (50% formamide [FA, AM9342, Thermo Fisher], 10% [w/v] dextran sulfate [D8906, Sigma-Aldrich] in 2X SSC [AM9763, Thermo Fisher]) for 15 min at 37°C. Primary probe libraries were diluted to a concentration of 100–300 ng/μL in 1X hybridization buffer and hybridized for 1–2 days at 37°C in a humidified chamber. After hybridization, cells were washed 2x with 50% FA in 2X SSC for 5 min at RT, followed by 3x with 2X SSC containing 0.2% Tween-20 (P2287, Sigma-Aldrich) and 2x with 2X SSC. Cells were incubated with 0.05 U/μL RNAse H (M0297S, NEB) for 20 min at 37°C in RNAse H buffer (NEB) to remove RNA-DNA hybrids. After washing 3x with 2X SSC, DNA bridges were hybridized to primary FISH probes to enable the common fluorescent readout of each library targeting a specific genomic locus or chromosome. To this end, DNA bridges were diluted to 100 nM in secondary hybridization buffer (20% Ethylene Carbonate (EC, E26258, Sigma-Aldrich), 2X SSC) and allowed to hybridize to primary probes in the sample for 20 min rocking at RT. Subsequently, cells were washed 3x with 30% FA in 2X SSC for 5 min each, rocking at RT, and then 2x with 2X SSC.

#### FISH protocol comparison

Cells from a mitotic shake-off were fixed, stained with DAPI and imaged on a spinning disk confocal. After the first round of imaging, the cells were prepared for FISH using non-denaturing or denaturing (denatured for 5 min with 0.1M HCl in 1X PBS, followed by incubation at 86 °C for 3 min during primary probe hybridization) conditions, stained with DAPI and the same cells were relocated and imaged. Mitotic cells (all stages from prophase to early telophase) were cropped manually from full fields of view of pre-treatment cells, then each pre- and post-treatment single cell image was registered in 3D using elastix with either Euler (rigid) registration or affine registration. The intensity of the pre- and post-treatment images was normalized by histogram matching, and the pixel-wise Pearson’s correlation coefficient was measured between the images using the binarized before-image as a mask.

#### SMC4-degradation and TSA treatment controls

To control for the efficient degradation of SMC4 or hyperacetylation by TSA treatment, HK-SMC4-mAID-Halo or HK WT cells were synchronized and treated as described above in “synchronization with targeted perturbation”, excluding the pre-synchronization using mitotic shake-off, as no increased enrichment of mitotic cells was required. SMC4-mAID-Halo cells were labelled using 100 nM Halo-TMR (Promega, G8251) for 10 min at 37°C prior to fixation. HK WT cells +/− TSA were immunostained against histone H3 acetylation using the primary rabbit antibody anti acetyl-histone H3 (Merck-Millipore, 06–599, dilution: 1:1,000), followed by a secondary hybridization using goat anti-rabbit AlexaFluor 488 (Molecular probes, A-11034, 1:1,000), as described above. Cells were stained with DAPI (0,5 ng/μl in 1X PBS for 5 min) and imaged on a Nikon TI-E2 microscope with Omicron lasers, a SR P-Apochromat IR AC 60× 1.27 NA water immersion objective, a CSU-W1 SoRa spinning disk unit and an Orca Fusion CMOS camera, operated using NIS Elements 5.2.02 (Nikon). 6–7 fields of view were sufficient to capture 40–60 mitotic cells in 3D with a pixel size of 227×227×500 nm in xyz and a field of view size of 261.4×261.4×25 μm. Cells were analyzed using a custom-written Python script. In brief, the chromosome’s/nuclei signal was segmented using the DAPI channel after a mild gaussian blur and small objects, as well as objects on the image borders, were removed. Mitotic and interphase cells were classified manually using napari. The resulting chromatin mask and labels were used to extract mean fluorescence intensities per cell. After background correction (empty regions in image), the mean fluorescence intensities per cell were multiplied with the segmented volume to yield integrated intensities, which were used to generate the plots.

#### Chromatin tracing

Chromatin tracing using LoopTrace was performed as described recently.^17,22^ FISH-treated cells were mounted on a Nikon TI-E2 microscope with Omicron lasers, a 100X 1.35 NA silicon oil immersion objective, a CSU-W1 SoRa spinning disk unit, a pentaband filter and an Orca Fusion CMOS camera, operated using NIS Elements 5.2.02 (Nikon). The Ibidi μ-Slide was connected to a custom-built automated fluidics robot based on a GRBL controlled CNC stage, as described in Beckwith et al.^17^ (see https://git.embl.de/grp-ellenberg/tracebot), to enable automated liquid handling for sequential imaging. Prior to sequential imaging, 25–35 3D stacks of DAPI-stained nuclei (405 nm excitation), Condensins (488 nm excitation, see immunofluorescence section) and fiducial beads (561 or 640 excitation) were acquired as reference images for cell classification. Images had a pixel size of 130 nm in xy and 300 nm in z and a total size of 149.76×149.76 μm in xy and covering a z-range of ~20 μm. After that, fluorescent imager probes targeting either a single genomic spot within each FISH library, or an entire library via a bridge probe, were sequentially hybridized through the automated liquid handling system and imaged. We performed dual-colour tracing with Cy3B- and Atto643-labeled 12 bp imager probes. Imager probes were stored in a 96 well plate on the CNC stage. In addition, the buffers for washing (10 % FA, 2X SSC), stripping (30% FA, 2X SSC) and imaging (56 mg/mL glucose oxidase (G7141, Sigma-Aldrich), 3.4 mg/mL catalase (C3155, Sigma-Aldrich), 1.5 mM Trolox (238813, Sigma-Aldrich), 10% Glucose, 50 mM Tris, 2X SSC pH 8.0), were stored in extra 3-well plates, covered in parafilm and mounted on the CNC stage. Imaging buffer was covered with a layer of light mineral oil (330779, Sigma-Aldrich) to prevent buffer degradation over the course of a 20-h imaging run. Imager strands and buffers were drawn with a syringe needle mounted in place of the CNC drill head. This needle was connected to the sample and a CPP1 peristaltic micropump (Jobst Technologies, Freiburg, Germany, flow rate of 1 mL/min at maximal speed) using 1 mm i.d. PTFE and silicone tubing (Bola, VWR), allowing to pull liquids out of the well plates and through the sample channel in an automated manner. Imager strands were sequentially hybridized for ~2 min at 20 nM in 5% EC 2X SSC, followed by 1 min incubation with washing buffer. After addition of imaging buffer, the same cells imaged in the reference stacks were imaged with same imaging dimensions in xyz in the 561 nm or 640 nm channels (100% laser power, 100 msec exposure time, triggered acquisition mode) to image Cy3B or Atto643-labelled imagers, respectively, alongside fiducial beads. After each round of imager strand hybridization and imaging, the imaged imager strands were stripped off using stripping buffer for 2 min and cells were washed again for 1 min using washing buffer.

#### 1 Mb and 10 Mb scale trace data processing

Processing of acquired 1 Mb and 10 Mb-scale tracing data was performed as described in Beckwith et al.^17^ with code available at https://git.embl.de/grp-ellenberg/looptrace. In brief, nd2 image files were converted to OME-ZARR format. Sequential images were registered by cross-correlation, and residual sub-pixel drift was corrected by calculating the mean offset of 3D gaussian centroids of segmented fiducial beads. Images were deconvolved using an experimental PSF extracted from registering and averaging 200 fiducial bead 3D images. Tracing regions were identified and segmented based on regional barcodes using an empirical intensity threshold. Each tracing region was background-corrected by subtraction of a blank frame, and up to 3 potential spots were identified per frame by local peak detection. Centroids of each spot were super-localized by fitting a 3D gaussian function. After filtering for poor fits, the fit closest to the median position of all high-quality unambiguous fits was retained for each frame. Chromatic aberration between Cy3B and Atto643 signals was corrected by least square fitting of the centroid of fiducial beads imaged in both channels, and traces were assigned to nuclei classified as “interphase”, “prophase”, “prometaphase” or “metaphase” based on chromosome organization of each nucleus.

#### Full chromosome trace data processing

Image conversion, registration and deconvolution were performed as for the higher resolution regions. To reduce the required number of tracing cycles, full chromosome sequential frames included the full chromosome regional barcode, 15 Mb segment barcodes and 30 spot barcodes repeating every 30 Mb (spanning 2 segments). Decoding the combined spot and segment identity identified the genomic coordinate of each spot. Full chromosome regions were segmented from regional barcodes using a random forest classifier (scikit-learn^55^) trained on 5–10 small foreground and background regions in one field of view. All potential peaks in the region were detected on background-corrected spot frames by local peak detection in scikit-image^56^ and fit by a 3D gaussian. Fits were quality controlled and corrected for chromatic aberration as above. To assign each fit to a 15 Mb segment, the intensity of each fit coordinate was measured in each potentially matching segment frame and assigned a z-score by subtracting the mean and dividing by the standard deviation of the peak intensity in all segment frames. To account for the potential presence of FISH signal from multiple chromosomes in the same region of interest, remaining ambiguous fits (i.e. more than one high-quality fit assigned to the same genomic coordinate) were assigned by spectral clustering based on spatial and genomic position into an increasing (1–5) number of clusters until most (75^th^ quantile) fits were unambiguously assigned to a chromosome trace. Remaining ambiguous fits were removed by selecting the fits with the highest z-score. Finally, traces were assigned to the cell cycle stage of their parent nuclei.

### QUANTIFICATION AND STATISTICAL ANALYSIS

#### Trace analysis

For analysis, 1 Mb and 10 Mb-scale fits were quality-controlled based on fit standard deviation and signal to background ratio, while whole chromosome fits were used directly. Median pairwise Euclidean distances were calculated for all 3D coordinates within a single trace and used to calculate distance scaling plots. A cut-off of minimum 30 points per trace was used. Scaling exponents were calculated by fitting tracing data at selected genomic intervals to a power-law function (y=ax^b^) using non-linear least squares fitting (scipy curve_fit). Median pairwise Euclidean distances were also used to compute trace metrics for the 1 Mb regions, with a minimum cut-off of 80 points. To assess potential compaction below the highest tracing resolution, we calculated the sum of sequential 3D distances in each trace (contour length). This measure indicated that no major compaction/de-compaction happened on length scales below the sampling resolution of our chromatin tracing (Figures S2B–S2D), validating that our 12 kb resolution sampled the major fine-scale folding changes during mitosis.

To compare tracing data with publicly available HiC data^11^ from prometaphase-arrested HeLa cells, median pairwise distance scaling values for Chr2q were inversed to units of nm^−1^, while P(s) curves were calculated by averaging balanced contact frequencies of 10 kb-binned HiC data from Chr2q across each genomic interval.

Compartments from available HiC data^52^ or tracing data were visualized as described previously.^57,58^ Contact frequencies/pairwise distances were normalized by their mean/median value at each genomic distance, and correlations coefficients were calculated $C_{ij}=Cov\left(row_{i},column_{j}\right)/\left(\sigma \left(row_{j}\right)*\sigma \left(col_{J}\right)\right)$. Compartment strength was plotted using available eigenvector-derived compartments scores matching the HiC data.^52^

Whole chromosome axes were estimated by a 20 Mb rolling mean, and chromosome radius was calculated by the distance from each point to the closest point on the axis. Axial scaling of whole prometa- and metaphase chromosomes were calculated by robust linear regression (Siegel slopes) of physical distance scaling data from 20–60 Mb genomic distances. Loop metrics for the 1 Mb scale were based on a 100 nm 3D distance cutoff to identify loop anchors spanning at least 30 kb genomic distance, and the relative position of loop anchors determined assignment to base loop, Z-loop or nested loops categories. Helicity of full chromosome traces was estimated by measuring the standard deviation of radial angles of points sampled at regular genomic intervals. 1 Mb and 10 Mb-scale traces were matched to their corresponding full chromosome traces by spatial proximity of their centroids. 3D nucleus and trace visualizations were done using mayavi after applying a rolling distance filter which removed spurious fits deviating more than 3 standard deviations from the median position of 11-point windows. Fluorescence images were displayed in napari.

#### Statistical analysis of DNA-trace metric data

Following Kruscal-Wallis test for overall significance, p-values were calculated using post-hoc pairwise test for multiple comparisons of mean rank sums calculated using scikit-posthoc_conover with the “holm” p value adjustment method.

#### Mitotic chromosome simulations

1D loop extrusion simulations were performed using a numpy/numba based framework as described previously,^17^ with loop extruder abundance and residence time set to physiological values for Condensins based on in vivo measurements in HeLa cells.^9,22^ Parameters unknown in vivo (Condensin extrusion rate and interaction between Condensin complexes) were empirically tested in pilot polymer simulations Simulation parameters are listed in Table S3. The simulations were run for 2,400 one second timesteps, corresponding to ~40 min mitosis from early prophase to metaphase.^9^ 3D polymer simulations were performed using the polychrom package which implements OpenMM polymer simulations.^59,60^ Simulations were run on Nvidia RTX 3090 GPUs on the EMBL HPC Cluster. Harmonic potential bonded neighbouring monomers and a harmonic potential with half bond length was used for loop extruder bonds. A polynomial repulsive force between non-neighbouring monomers was used to ensure self-avoidance of the polymer. No boundary conditions were used. The simulated polymers consisted of 100,000 monomers. The simulations were equilibrated from a random walk starting conformation for 100,000 simulation steps and scaled to experimental 12 kb resolution ΔSMC4 mitotic tracing data, giving scaled bond-lengths of 12 nm. Dynamic mitotic simulations were performed by first equilibrating the random walk starting configuration for 100,000 simulation steps, then applying and updating loop extruder bond positions sampled from the 1D simulations every 5^th^ loop extrusion step and equilibrated for 20,000 steps, giving a total of 2,500,000 simulation steps for mitotic simulations (~15 min GPU run time per chromosome). In simulations of TSA-treated chromosomes, the maximum repulsive potential was increased 5-fold and repulsion radius increased from 1.05 bond lengths to 1.5. The resulting polymer conformations with 1 kb monomers were sampled to match the experimental sampling of 1 Mb, 200 kb or 12 kb before analysis. Regular helical chromosomes with 12 Mb pitch were mathematically generated, and normally distributed noise with a μ=0, σ=200 nm was added to x, y, and z positions of regular helices to simulate noisy helical chromosomes.

## Supplementary Material

1 Figure S1. Non-denaturing FISH enables multiscale tracing of mitotic chromatin, related to Figure 1
(A) Synchronization schemes for cells enriched in prophase/prometaphase (left) and metaphase (right).
(B) Representative images (left) and pixel-wise Pearson correlation coefficient (right) of PFA-fixed mitotic cells labeled with DAPI and imaged before (magenta) and after (green) treatment with heat-denaturation FISH, non-denaturing/RASER-FISH, or simulated FISH (non-denaturing FISH without library hybridization, control). Two registration algorithms were tested, with affine registration showing improved overlay accuracy as uniform scaling was better compensated. Data from one experiment with *n* = 92 cells (denaturing FISH), *n* = 78 cells (non-denaturing FISH), and *n* = 72 cells (control). Median, quartiles, and whiskers are shown in the plots.
(C) Trace completeness as measured by the percentage of possible spots detected per trace across different libraries in interphase and metaphase. Data from 13,455 traces in 1,712 cells in 5 independent experiments. HeLa cells contain a truncated copy of chr2, which reduces the completeness of whole chr2 traces, compared with other regions. Median, quartiles, and whiskers (1.5 times interquartile range [IQR]) are shown in the plots.
(D) Tracing precision as measured by the absolute deviation in fit position after drift correction when the same genomic position was re-labeled in a different imaging cycle. Data from *n* = 373 (interphase, 12 kb), *n* = 395 (metaphase, 12 kb), *n* = 198 (interphase, 30 kb), and *n* = 250 (metaphase, 30 kb) traces in one representative experiment of 2 independent experiments. Median, quartiles, and whiskers are shown in the plots.
(E) Median pairwise distance maps of a 1.2-Mb region (chr2:191,110,000–192,309,940) traced at 12-kb resolution. Number of traces as indicated from a total of 666 cells in 2 independent experiments.
(F) Median pairwise distance maps of a 1.2-Mb region (chr5:149,500,723–150,699,922) traced at 12-kb resolution. Number of traces as indicated from a total of 874 cells in 3 independent experiments.
(G) Length and width of mitotic chromosomes, estimated by length of a 20-Mb rolling average axis and median distance of each point to the closest rolling average from chr2 full chromosome library (1-Mb resolution). Data from 117 interphase, 19 prophase, 168 prometaphase, and 212 metaphase chromosomes from 3 independent experiments. Median, quartiles, and whiskers are shown in the plots.
(H) Data corresponding to (G), for chr14. Data from 112 interphase, 21 prophase, 292 prometaphase, and 273 metaphase chromosomes from 3 independent experiments.

2 Figure S2. Chromatin trace-metric analysis reveals mitosis-specific signatures, related to Figure 2
(A) Exemplary pairwise distance map showcasing the classification of loops. Nested loops (magenta) emerge when the bases of two loops (green) merge to form a loop contact with the size of the two base loops. Z-loops (cyan) overlap with each other partially.
(B) Additional trace metrics for chr5:149,500,723–150,699,962. Elongation indicates the ratio of minor to major axis of an ellipsoid fit to the trace, while contour length measures the cumulative point-to-point length of the trace. The similar contour length for the different phases indicates that the other structural metrics that change during mitosis are well sampled at this genomic resolution. Data from *n* = 278 (510), *n* = 22 (44), *n* = 124 (297), and *n* = 152 (398) interphase, prophase, prometaphase, and metaphase cells (traces) from 3 independent experiments. Median, quartiles, and whiskers are shown in the plots.
(C) Trace metrics for high-resolution tracing in chr2:191,110,000–192,309,940. Data from *n* = 171 (246), *n* = 13 (19), *n* = 100 (180), and *n* = 134 (248) interphase, prophase, prometaphase, and metaphase cells (traces) from 2 independent experiments. Median, quartiles, and whiskers are shown in the plots.
(D) Trace metrics for high-resolution tracing in chr14:50,923,646–52,104,342. Data from *n* = 136 (189), *n* = 9 (15), *n* = 94 (160), and *n* = 118 (210) interphase, prophase, prometaphase, and metaphase cells (traces) from one experiment. Median, quartiles, and whiskers are shown in the plots.
(E) Representative examples of a simulated regular helix (left), a regular helix with added Gaussian noise (center) and experimental data from chr2q (right), and plotted estimation of helical regularity. Helical regularity was measured by the standard deviation in radial angle between points separated by different genomic intervals. The radial angle was measured, compared with a 20-Mb rolling average axis. Local minima in the standard deviation indicating helical regularity are indicated by arrows. Data from 100 generated regular or noisy helices and the 100 best-resolved chr2q traces from 3 independent experiments. Mean and 95% confidence interval (estimated by bootstrapping) shown.

3 Figure S3. Multiscale chromatin traces reveal mitosis-specific genome scaling behavior, related to Figure 3
(A) Pairwise distance scaling plots for chr2:191,110,000–192,309,940 (12-kb resolution). Data from *n* = 711 (1,280) cells (traces), 2 independent experiments. Plots show median ± standard error of the mean.
(B) Pairwise distance scaling plots for chr14:50,923,646–52,104,342 (12-kb resolution), chr14:45,200,003–56,429,971 (200-kb resolution), and chr14:20,000,036–105,029,664 (1-Mb resolution). Data from *n* = 677 (1,243) cells (traces), 2 independent experiments; *n* = 645 (1,201) cells (traces), 2 independent experiments; and *n* = 543 (1,039) cells (traces), 2 independent experiments, respectively. Plots show median ± standard error of the mean. As the scaling data become very sparse at maximal genomic trace distance, the scaling plots were cropped to 1, 10, and 80 Mb, respectively.
(C) Pairwise distance scaling plots for chr18:50,000,077–62,829,816 (200-kb resolution). Data from *n* = 633 (1,134) cells (traces), 2 independent experiments. Plots show median ± standard error of the mean.
(D) Distance scaling plots on log-log scale from chr5 (1-Mb scale), chr2 (10-Mb scale), and chr2q (100-Mb scale), corresponding to Figure 3. For the 1- and 10-Mb scales, power law fits below and above 100 kb and 3 Mb, respectively, are shown with dashed lines, and fit exponents are shown in the table below the graph. No fit was performed on full chromosome data.
(E) Top row: inverted median distance scaling for chr2q (left column) and chr14q (right column) in metaphase HeLa cells sampled at 1-Mb resolution. Middle row: HiC P(s) scaling plots from chr2q and chr14q from prometaphase-arrested HeLa cells (Gibcus et al.,^11^ GEO: GSM2745897), downsampled by averaging to 1-Mb resolution. The dashed line is placed at 8 Mb and indicates the approximate position of the scaling dip (visualized as a bump in this representation). Bottom row: scatterplot of log-transformed HiC frequencies against pairwise distances for chr2q and chr14q. Linear fits to the data below and above 1,000 nm are overlaid. See Figure 3C and (B) for description of the tracing dataset.
(F) Exemplary fluorescent micrographs of metaphase/anaphase cells used for chromatin tracing. Reconstructed 3D traces (multi-colored) from the library targeting whole chr2. For better comparison of the anaphase chromosome trace with other presented trace data in this manuscript, the same metaphase chr2 trace as in Figure 6C is displayed.
(G–I) Pairwise distance scaling plots showing median pairwise distance ± standard error of the mean for a high-resolution region (chr5, G), an intermediate scale (chr2, H), and whole chr2 (I) and corresponding pairwise distance scaling plots. Data from (G) *n* = 227 (644) and *n* = 61 (123), (H) *n* = 198 (346) and *n* = 40 (71), and (I) *n* = 208 (357) and *n* = 55 (80) metaphase and anaphase cells (traces) measured in 3 independent experiments, respectively.
(J) Trace metrics calculated for the high-resolution (12 kb) 1.2-Mb region on chr5 (149,500,723–150,699,962). Trace metrics were calculated from traces that were more than 80% complete. Data from *n* = 152 (398) and *n* = 61 (87) metaphase and anaphase cells (traces) from 3 independent experiments. Median, quartiles, and whiskers are shown in the plots.
(K) Length, width, and distance scaling slope of WT metaphase and anaphase chr2, estimated by length of a 20-Mb rolling average axis and median distance of each point to the closest rolling average point from chr2 whole-chromosome traces (1-Mb resolution). Data from 137 (245) metaphase cells (traces) and 36 (60) anaphase cells (traces), 3 independent experiments. Median, quartiles, and whiskers are shown in the plots.

4 Figure S4. Extended analysis of condensin-depleted mitotic chromosomes, related to Figure 4
(A)Experimental scheme for condensin-depleted mitotic chromosomes, using the HK SMC4-mAID cell line.^28^
(B) Fluorescent micrographs of HK DSMC4-mAID-Halo metaphase cells in WT condition or after 3 h of pre-mitotic SMC4 degradation. Cells were stained with Halo-TMR ligand to visualize SMC4. Scale bar, 5 mm.
(C) After 3 h of pre-mitotic DSMC4-depletion, cells were completely devoid of SMC4. *n*WT = 52, *n*ΔSMC4 = 44. Median, quartiles, and whiskers are shown in the plots.
(D) Trace metric from chr5:149,500,723–150,699,962 (1.2 Mb, 12-kb resolution) for WT and ΔSMC4 cells. Data from 152 (398) WT cells (traces), 3 independent experiments, and 153 (265) ΔSMC4 cells, 2 independent experiments. Median, quartiles, and whiskers are shown in the plots.
(E) Distance scaling plots for chr14 10 Mb and whole-chromosome scales. Data from chr14, 10 Mb: 218 (434) WT cells (traces), 2 independent experiments, and 217 (419) ΔSMC4 cells (traces), 2 independent experiments; chr14, whole: 174 (357) WT cells (traces), 2 independent experiments, and 78 (146) ΔSMC4 cells (traces), one experiment. As the scaling data become very sparse at maximal genomic trace distance, the scaling plots were cropped to 10 and 80 Mb, respectively.
(F) Distance scaling plots on log-log scale from chr5 (1-Mb scale), chr2 (10-Mb scale), and chr2q (100-Mb scale), corresponding to Figure 4D. For the 1- and 10-Mb scales, power law fits below and above 100 kb and 3 Mb, respectively, are shown with dashed lines, and fit exponents are shown in the table below the graph.
(G) As in (F), with data from chr14 (10-Mb scale) and chr14q (100-Mb scale), corresponding to the data shown in (E).
(H and I) Representation of A/B compartments from available HiC data (4DN dataset 4DNFICCAQVVF, left column), highlighted by calculating distancenormalized contact frequencies (center column) and their correlation matrices (right column). Compartment scoring by eigenvector analysis (4DN dataset 4DNFI81BFOEQ) is shown below the matrices. A region with a strong compartment score is highlighted by a dashed magenta box.
(J and K) Representation of DNA tracing data from the regions indicated in (H) and (I) from HeLa WT cells in interphase (top row), metaphase (center row), or in metaphase after condensin depletion (lower row). Data represented as median pairwise distances (left column), normalized distances (center column), or correlation matrices (right columns). See STAR Methods for a detailed description of the representations and Figures 3B and S3B for description of the tracing dataset.

5 Figure S5. A data-constrained dynamic polymer model of condensin-driven loop extrusion predicts mitotic chromosome organization, related to Figure 5
(A) Distance scaling plot of an unconstrained polymer to experimental DSMC4 metaphase high-resolution tracing data (12-kb resolution, chr5) gave 12 nm/kb monomer and 1 k_b_T repulsive potential as optimal polymer simulation parameters. Experimental data from 212 (608) DSMC4 cells (traces), 2 independent experiments.
(B–D) Distance scaling plots and histograms of condensin I (magenta) and condensin II (green) loop lengths resulting from alternative loop extrusion models, including (B) altered extrusion speed assuming one-sided extrusion and no stalling, (C) one- and two-sided extrusion at 4 ± 2 kbps with full stalling, and (D) altered extrusion speed distributions assuming one-sided extrusion and no stalling. Simulation positions were sampled as experimental data (100-kb spots, 1-Mb resolution) 10 times per 100-Mb chromosome with offset genomic positions. Simulated data from 10 to 20 dynamically simulated metaphase (40 min) chromosomes per condition. Experimental data from chr2q, 100 Mb: *n* =2 12 (686) cell (traces), 3 independent experiments.
(E) Distance scaling plots of simulated chromosomes, assuming one-sided extrusion at 4 ± 2 kbps and no stalling, and corresponding experimental data. Simulated traces were spatially sampled as experimental data (12-kb tiled probes, 30-kb probes with 200-kb resolution, and 100-kb probes with 1-Mb resolution). Sampling time points were 0, 10, 32, and 40 min for interphase, prophase, prometaphase, and metaphase, respectively. Simulated data from *n* = 20 dynamically simulated 100-Mb chromosomes. Experimental data from HeLa WT cells; see Figures 3 and S3 for detailed information.
(F) Ground-truth positions of condensin I (magenta) and condensin II (green) in simulated chromatin (2-kb sampling in light gray, 1-Mb rolling average in dark gray), corresponding to the example in Figure 5A.
(G) Radial distribution of condensin I, condensin II, and chromatin in simulated 100-Mb chromosomes. Radial distances were calculated compared with a 20-Mb rolling average axis for 20 dynamically simulated chromosomes sampled at metaphase (40 min), assuming one-sided extrusion at 4 ± 2 kbps and no condensin stalling.

6 Figure S6. Histone hyperacetylation leads to mitotic chromosome decompaction while preserving condensin-driven features, related to Figure 6
(A) Experiment scheme for the generation of hyperacetylated mitotic chromosomes using TSA treatment during interphase.
(B) Fluorescent micrographs of HK WT metaphase cells in WT condition or after 5 h of pre-mitotic TSA treatment. Cells were stained with an anti-histone 3 acetyl antibody to visualize histone acetylation levels. Scale bar, 5 μm.
(C) Scatterplot shows a positive correlation between histone acetylation levels and chromosome volume. *n*WT = 59, *n*ΔSMC4 = 58.
(D) Trace metric from chr5:149,500,723–150,699,962 (1.2 Mb, 12-kb resolution) for WT and TSA-treated cells. Data from 152 (398) WT cells (traces), 3 independent experiments, and 67 (168) TSA-treated cells (traces), 2 independent experiments. Median, quartiles, and whiskers are shown in the plots.
(E) Distance scaling plots from chr14, 10 Mb and whole-chromosome scales for WT, DSMC4, and TSA-treated cells at metaphase. Chr14, 10 Mb: 69 (133) TSA-treated cells (traces), 2 independent experiments; chr14, whole: 15 (30) TSA-treated cells (traces), one experiment. See Figure S4 for details on WT and DSMC4 data. As the scaling data become very sparse at maximal genomic trace distance, the scaling plots were cropped to 10 and 80 Mb, respectively.
(F) Distance scaling plots on log-log scale from chr5 (1-Mb scale), chr2 (10-Mb scale), and chr2q (100-Mb scale), corresponding to Figure 6D. For the 1- and 10-Mb scales, power law fits below and above 100 kb and 3 Mb, respectively, are shown with dashed lines, and fit exponents are shown in the table below the graph.
(G) As in (F), with data from chr14 (10-Mb scale) and chr14q (100-Mb scale), corresponding to the data shown in (E).
(H) Length and width of WT and TSA-treated metaphase chr2 and chr14, estimated by length of a 20-Mb rolling average axis and median distance of each point to the closest rolling average point from chr2 whole-chromosome traces (1-Mb resolution). Data from 130 (212) WT cells (traces), 3 independent experiments, and 19 (31) TSA-treated cells (traces), 2 independent experiments. Median, quartiles, and whiskers are shown in the plots.
(I) Length and width of 100-Mb simulated chromosomes under WT and TSA-treated conditions, and corresponding experimental data from chr2 q-arm (100 Mb). Data from 20 dynamically simulated 100-Mb chromosomes per condition, sampled at metaphase (40 min). Experimental data detailed in (E). Median, quartiles, and whiskers are shown in the plots.

7

8

9

10

Supplemental information can be found online at https://doi.org/10.1016/j.cell.2025.02.028.

## Figures and Tables

**Table:** KEY RESOURCES TABLE

REAGENT or RESOURCE	SOURCE	IDENTIFIER
Antibodies		
Goat anti-rabbit IgG, Alexa Fluor^™^ 488	Thermo Fisher Scientific	Cat# A-11034 (Lot# 1616933); RRID: AB_2576217
Rabbit Polyclonal SMC2 antibody	Abcam	Cat# ab10412; RRID: AB_2192486
Chemicals, peptides, and recombinant proteins		
4’,6-diamidino-2-phenylindole (DAPI)	Sigma Aldrich	D9542
5-Bromo-2’-deoxycytidine (BrdC)	Santa Cruz Biotechnology	sc-284555
5-Bromo-2′-deoxyuridine (BrdU)	Sigma Aldrich	B5002
5-Phenyl-indole-3-acetic acid (5-Ph-IAA)	BioAcademia	30–003
Atto643-alkyne	Atto-Tec	AD 643–141
Bis(NHS)PEG5	Sigma Aldrich	803537
Catalase from bovine liver	Sigma Aldrich	C3155
Cy3B-alkyne	AAT Bioquest	ABD-944
Dextran sulfate sodium salt from Leuconostoc spp.	Sigma Aldrich	D8906
Ethylene Carbonate	Sigma Aldrich	E26258
Exonuclease III	New England Biolabs	M0206
Formamide	Thermo Fisher Scientific	AM9342
Glucose Oxidase from Aspergillus niger	Sigma Aldrich	G7141
Mineral oil, light	Sigma Aldrich	330779
Poly-L-lysine	Sigma Aldrich	P8920
RNAse H	New England Biolabs	M0297S
RO-3306	Sigma Aldrich	SML0569
SSC Buffer 20x	Thermo Fisher Scientific	AM9763
Trichostatin A	Sigma Aldrich	T8552
Trolox	Sigma Aldrich	238813
Critical commercial assays		
ClickTech Oligo Link Kit	Baseclick GmbH	BCK-OL-L
Deposited data		
Processed DNA-tracing data	this work	Figshare: https://doi.org/10.6084/m9.figshare.27003022
HiC contact matrix, HeLa S3, prometaphase-arrested	Gibcus et al.^11^	GEO: GSM2745897
HiC contact matrix, Hela S3, unsynchronized	Akgol Oksuz et al.^52^	4DNFICCAQVVF
HiC, compartment calls table, Hela S3, unsynchronized	Akgol Oksuz et al.^52^	4DNFI81BFOEQ
Experimental models: Cell lines		
HeLa Kyoto SMC4-mAID-Halo, AAVS1-OsTir1 F74G-SNAP	Schneider et al.^28^	N/A
HeLa Kyoto wild type	S. Narumiya (Kyoto University, Kyoto, Japan)	RRID: CVCL_1922
Software and algorithms		
LoopTrace Code	Beckwith et al.^17^; this work	https://git.embl.de/grp-ellenberg/looptrace
Microfluidics robot Code	Beckwith et al.^17^	https://git.embl.de/grp-ellenberg/tracebot
Code for trace analysis and visualization	this work	Figshare: https://doi.org/10.6084/m9.figshare.27003022
Other		
0.1 μm Tetraspec beads	Thermo Fisher	T7279
Ibidi glass-bottom μ-slide	Ibidi	80607
Lamp TUV 15W G15T8 (254 nm)	Philips	308643
UV Stratalinker 2400	Stratagene	021561

## Highlights

- 3D DNA tracing resolves the internal architecture of mitotic chromosomes
- Chromosomal rod shape emerges without a contiguous scaffold or regular handed helix
- Condensin traversal enables extrusion of overlapping loops up to several megabases
- Self-repulsion of overlapping loops generates mitotic chromosomal rod shape
