# Supplementary figures and images for: Nanoscale DNA tracing reveals the self-organization mechanism of mitotic chromosomes
Source: Cell. Author manuscript; Available in PMC 2025 Jun 2. (PMC12127698; doi:10.1016/j.cell.2025.02.028)

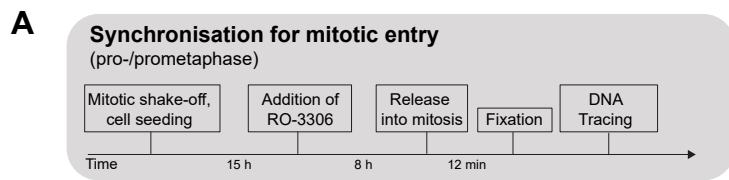

**Synchronisation for metaphase stage**  
(suitable for all mitotic stages except prophase)

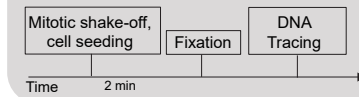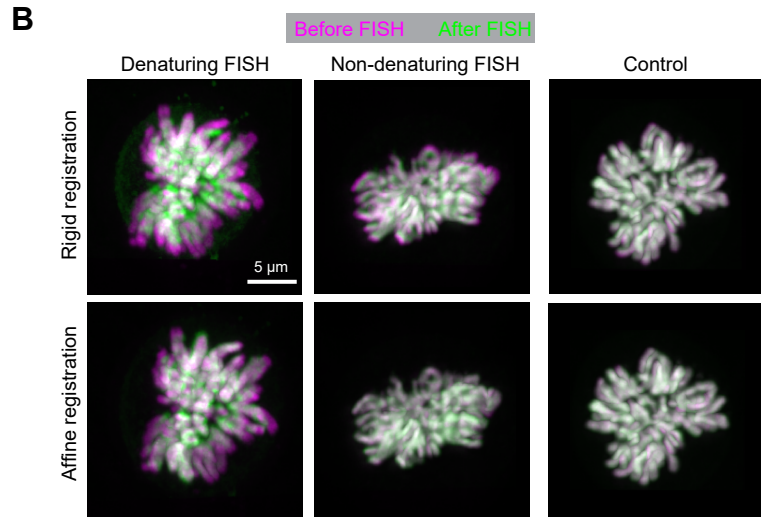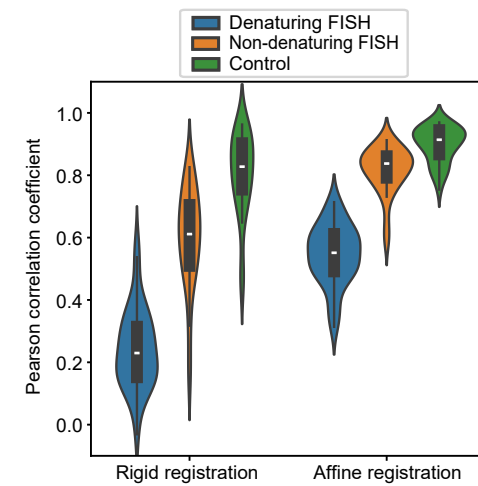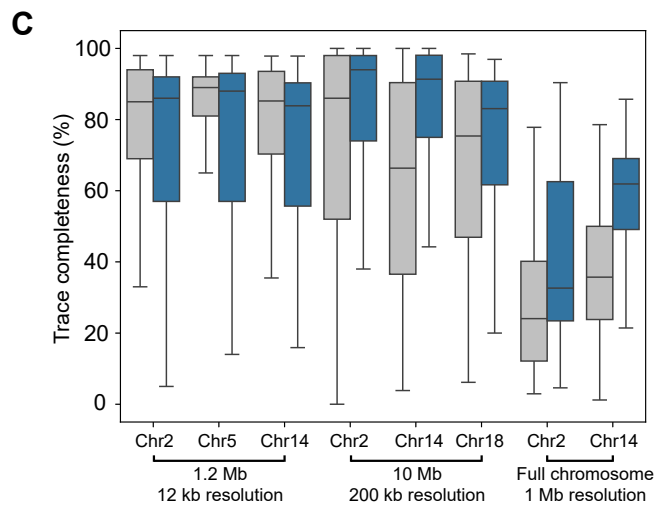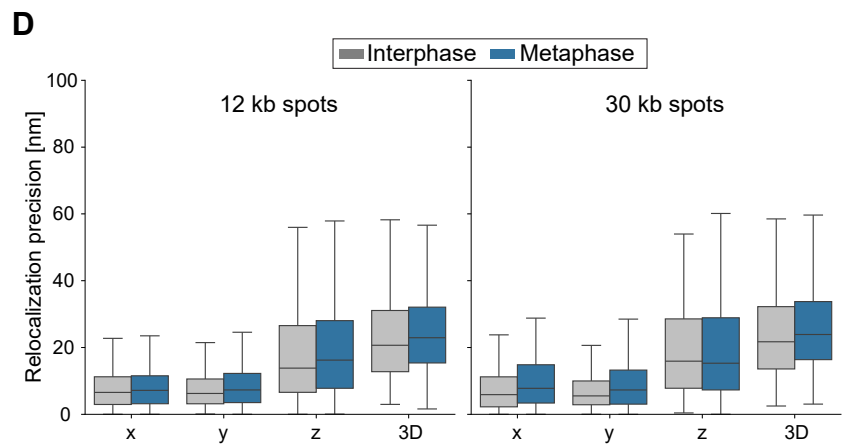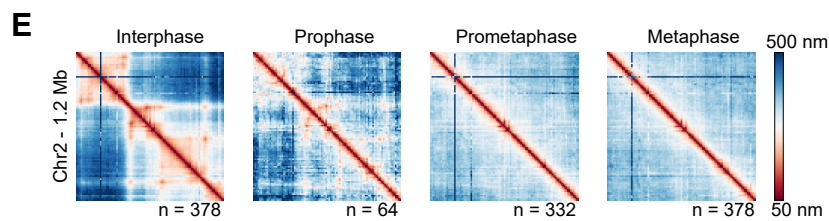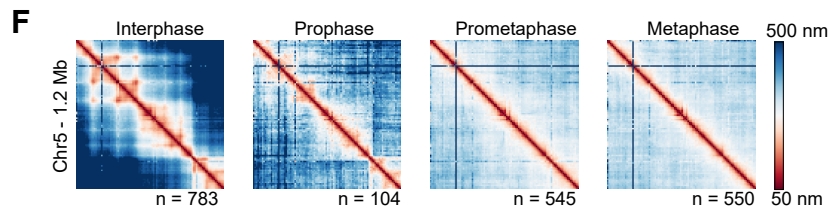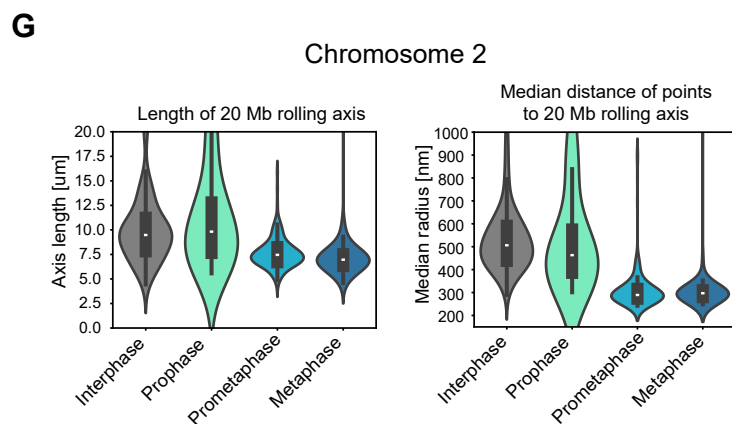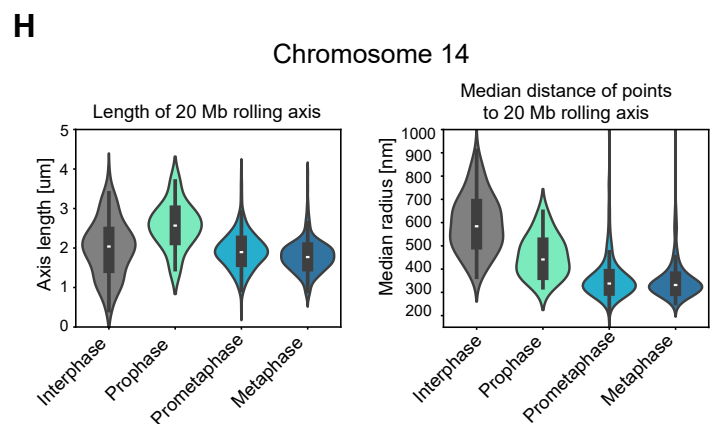

Supplement: 1 — Figure S1. Non-denaturing FISH enables multiscale tracing of mitotic chromatin, related to Figure 1 (A) Synchronization schemes for cells enriched in prophase/prometaphase (left) and metaphase (right). (B) Representative images (left) and pixel-wise Pearson correlation coefficient (right) of PFA-fixed mitotic cells labeled with DAPI and imaged before (magenta) and after (green) treatment with heat-denaturation FISH, non-denaturing/RASER-FISH, or simulated FISH (non-denaturing FISH without library hybridization, control). Two registration algorithms were tested, with affine registration showing improved overlay accuracy as uniform scaling was better compensated. Data from one experiment with n = 92 cells (denaturing FISH), n = 78 cells (non-denaturing FISH), and n = 72 cells (control). Median, quartiles, and whiskers are shown in the plots. (C) Trace completeness as measured by the percentage of possible spots detected per trace across different libraries in interphase and metaphase. Data from 13,455 traces in 1,712 cells in 5 independent experiments. HeLa cells contain a truncated copy of chr2, which reduces the completeness of whole chr2 traces, compared with other regions. Median, quartiles, and whiskers (1.5 times interquartile range [IQR]) are shown in the plots. (D) Tracing precision as measured by the absolute deviation in fit position after drift correction when the same genomic position was re-labeled in a different imaging cycle. Data from n = 373 (interphase, 12 kb), n = 395 (metaphase, 12 kb), n = 198 (interphase, 30 kb), and n = 250 (metaphase, 30 kb) traces in one representative experiment of 2 independent experiments. Median, quartiles, and whiskers are shown in the plots. (E) Median pairwise distance maps of a 1.2-Mb region (chr2:191,110,000–192,309,940) traced at 12-kb resolution. Number of traces as indicated from a total of 666 cells in 2 independent experiments. (F) Median pairwise distance maps of a 1.2-Mb region (chr5:149,500,723–150,699,922) tr [file NIHMS2067674-supplement-1.pdf]

**A**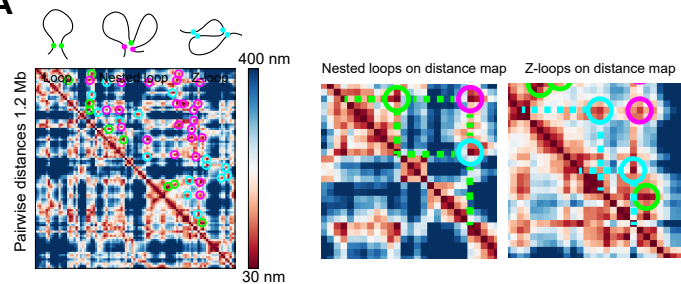**B**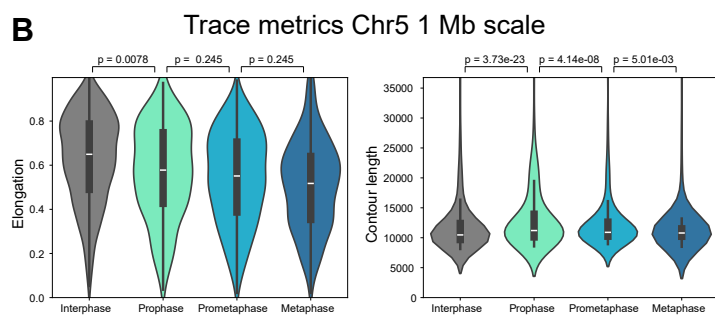**C**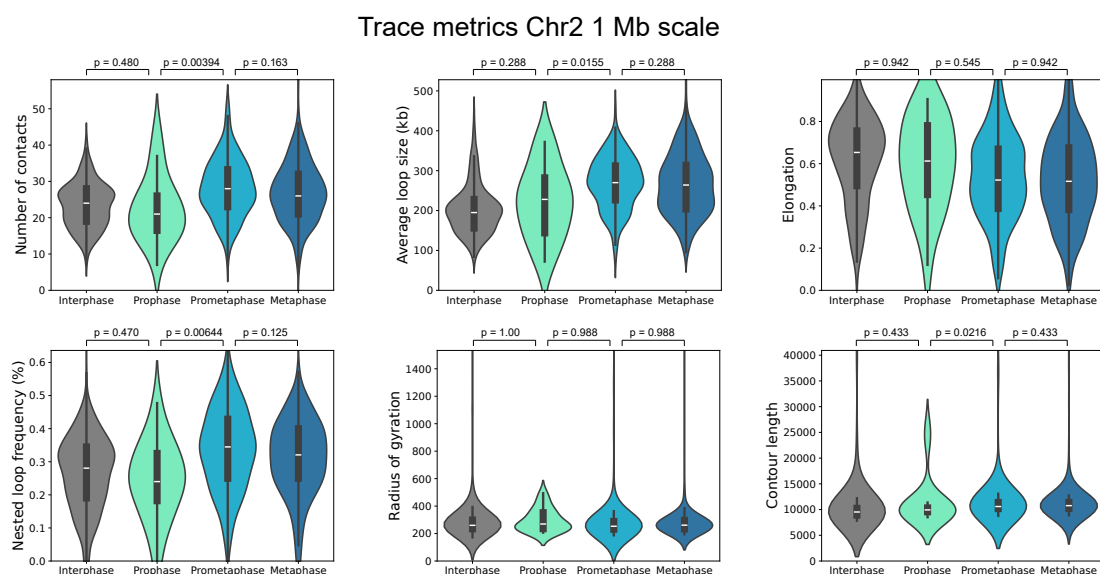**D**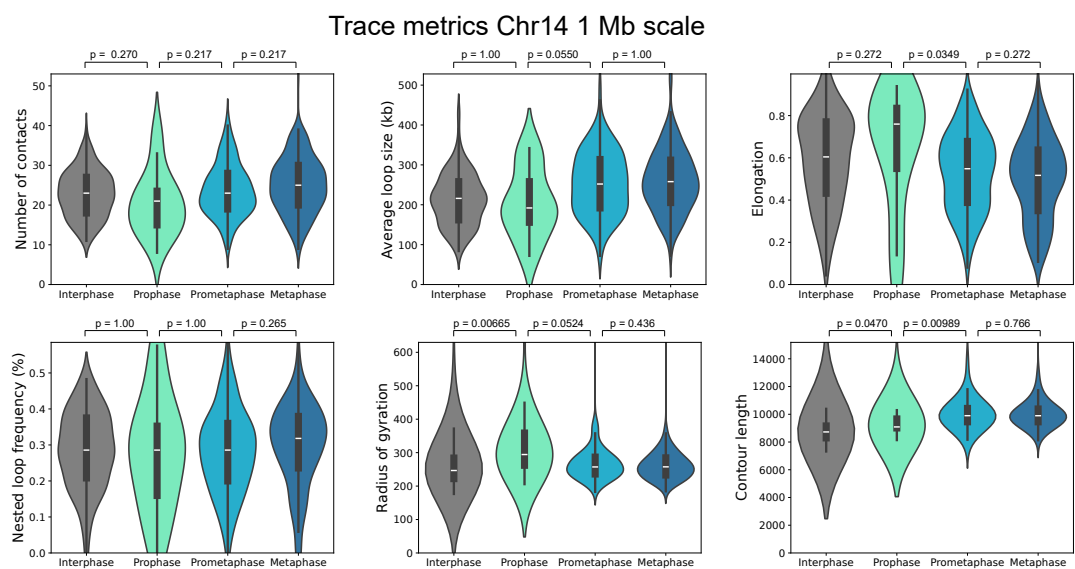**E**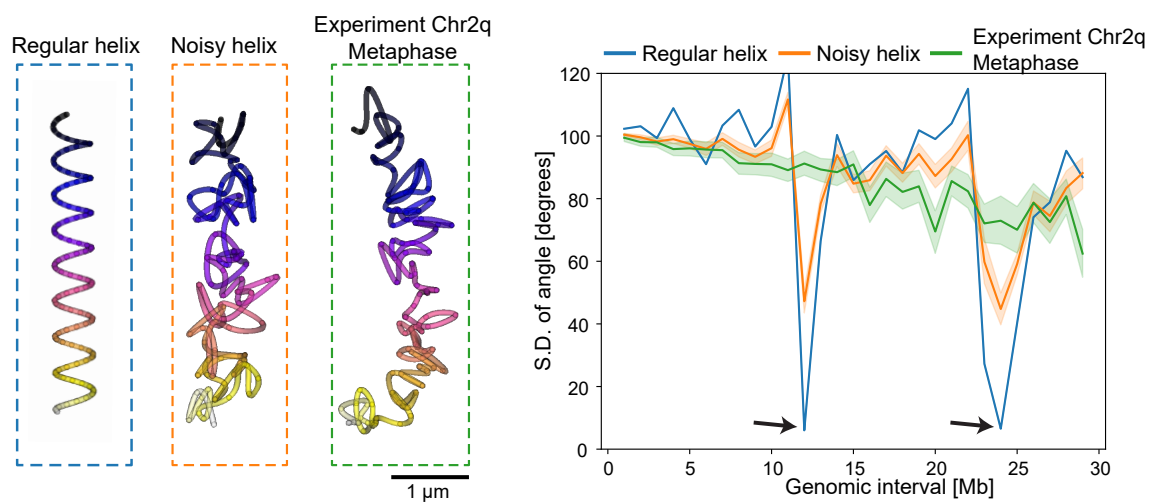

Supplement: 2 — Figure S2. Chromatin trace-metric analysis reveals mitosis-specific signatures, related to Figure 2 (A) Exemplary pairwise distance map showcasing the classification of loops. Nested loops (magenta) emerge when the bases of two loops (green) merge to form a loop contact with the size of the two base loops. Z-loops (cyan) overlap with each other partially. (B) Additional trace metrics for chr5:149,500,723–150,699,962. Elongation indicates the ratio of minor to major axis of an ellipsoid fit to the trace, while contour length measures the cumulative point-to-point length of the trace. The similar contour length for the different phases indicates that the other structural metrics that change during mitosis are well sampled at this genomic resolution. Data from n = 278 (510), n = 22 (44), n = 124 (297), and n = 152 (398) interphase, prophase, prometaphase, and metaphase cells (traces) from 3 independent experiments. Median, quartiles, and whiskers are shown in the plots. (C) Trace metrics for high-resolution tracing in chr2:191,110,000–192,309,940. Data from n = 171 (246), n = 13 (19), n = 100 (180), and n = 134 (248) interphase, prophase, prometaphase, and metaphase cells (traces) from 2 independent experiments. Median, quartiles, and whiskers are shown in the plots. (D) Trace metrics for high-resolution tracing in chr14:50,923,646–52,104,342. Data from n = 136 (189), n = 9 (15), n = 94 (160), and n = 118 (210) interphase, prophase, prometaphase, and metaphase cells (traces) from one experiment. Median, quartiles, and whiskers are shown in the plots. (E) Representative examples of a simulated regular helix (left), a regular helix with added Gaussian noise (center) and experimental data from chr2q (right), and plotted estimation of helical regularity. Helical regularity was measured by the standard deviation in radial angle between points separated by different genomic intervals. The radial angle was measured, compared with a 20-Mb rolling average axis. Local minima in [file NIHMS2067674-supplement-2.pdf]

**A**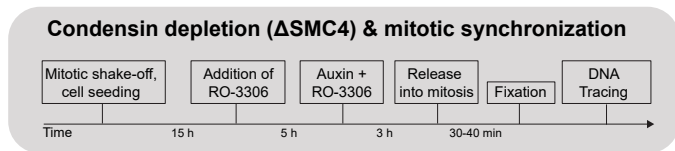**B**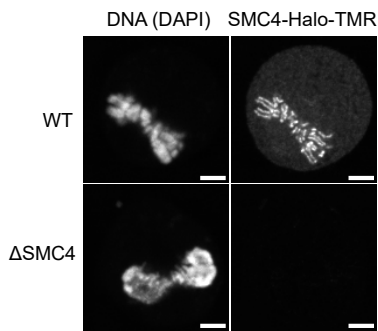**C**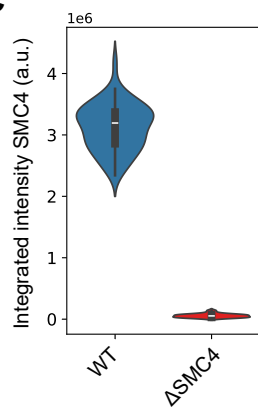**D**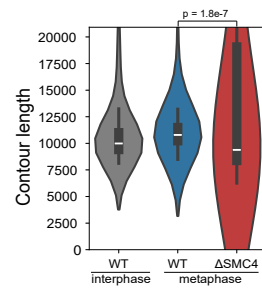**E**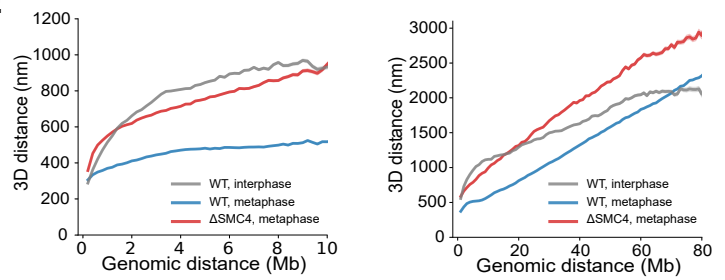**F**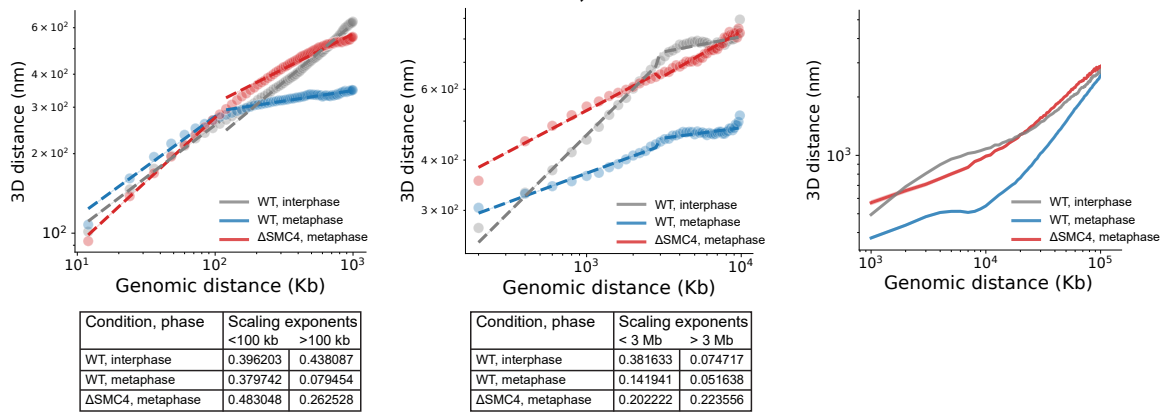**G**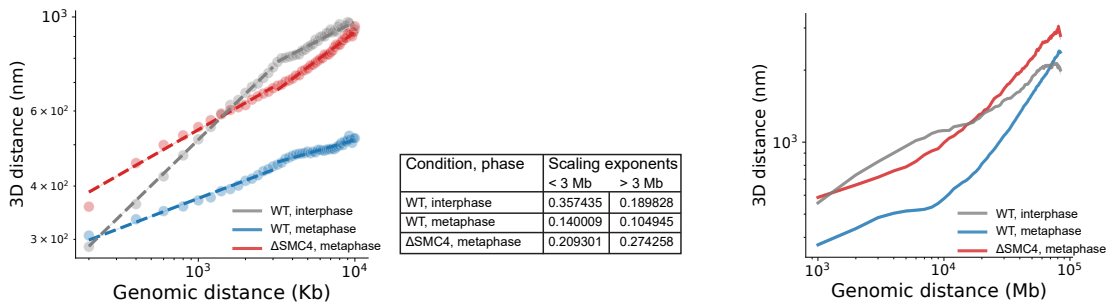**H**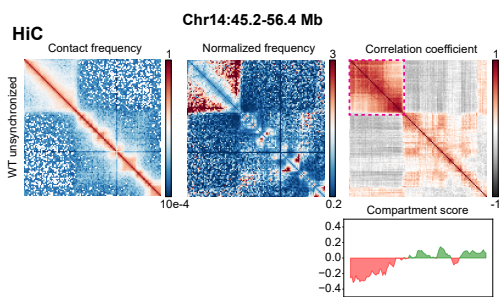**I**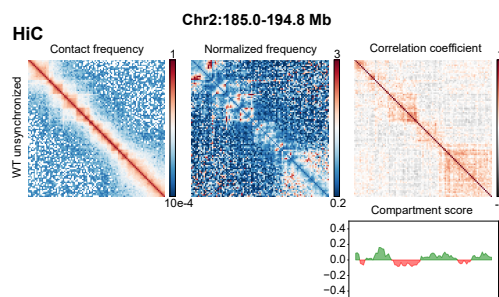**J**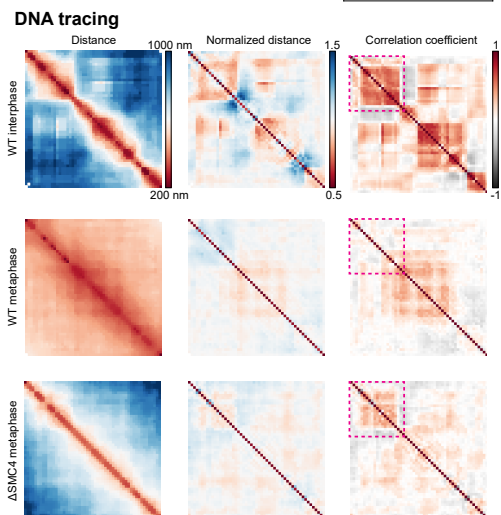**K**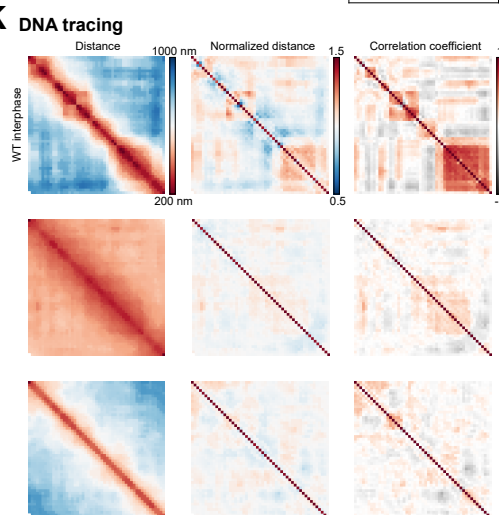

Supplement: 4 — Figure S4. Extended analysis of condensin-depleted mitotic chromosomes, related to Figure 4 (A)Experimental scheme for condensin-depleted mitotic chromosomes, using the HK SMC4-mAID cell line.28 (B) Fluorescent micrographs of HK DSMC4-mAID-Halo metaphase cells in WT condition or after 3 h of pre-mitotic SMC4 degradation. Cells were stained with Halo-TMR ligand to visualize SMC4. Scale bar, 5 mm. (C) After 3 h of pre-mitotic DSMC4-depletion, cells were completely devoid of SMC4. nWT = 52, nΔSMC4 = 44. Median, quartiles, and whiskers are shown in the plots. (D) Trace metric from chr5:149,500,723–150,699,962 (1.2 Mb, 12-kb resolution) for WT and ΔSMC4 cells. Data from 152 (398) WT cells (traces), 3 independent experiments, and 153 (265) ΔSMC4 cells, 2 independent experiments. Median, quartiles, and whiskers are shown in the plots. (E) Distance scaling plots for chr14 10 Mb and whole-chromosome scales. Data from chr14, 10 Mb: 218 (434) WT cells (traces), 2 independent experiments, and 217 (419) ΔSMC4 cells (traces), 2 independent experiments; chr14, whole: 174 (357) WT cells (traces), 2 independent experiments, and 78 (146) ΔSMC4 cells (traces), one experiment. As the scaling data become very sparse at maximal genomic trace distance, the scaling plots were cropped to 10 and 80 Mb, respectively. (F) Distance scaling plots on log-log scale from chr5 (1-Mb scale), chr2 (10-Mb scale), and chr2q (100-Mb scale), corresponding to Figure 4D. For the 1- and 10-Mb scales, power law fits below and above 100 kb and 3 Mb, respectively, are shown with dashed lines, and fit exponents are shown in the table below the graph. (G) As in (F), with data from chr14 (10-Mb scale) and chr14q (100-Mb scale), corresponding to the data shown in (E). (H and I) Representation of A/B compartments from available HiC data (4DN dataset 4DNFICCAQVVF, left column), highlighted by calculating distancenormalized contact frequencies (center column) and their correlation matrices (right column). Compartment [file NIHMS2067674-supplement-4.pdf]

**A**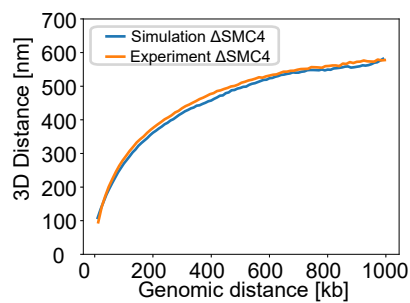**B**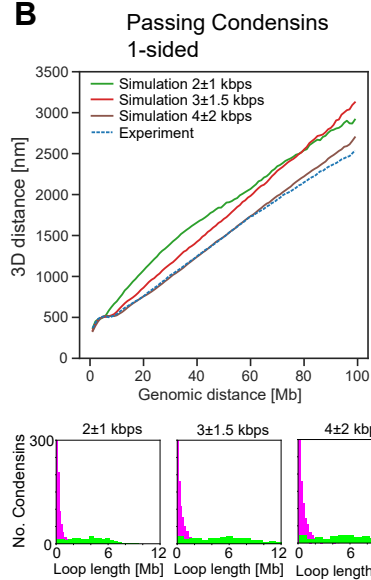**C**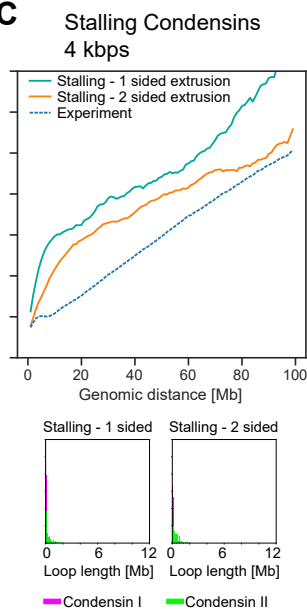**D**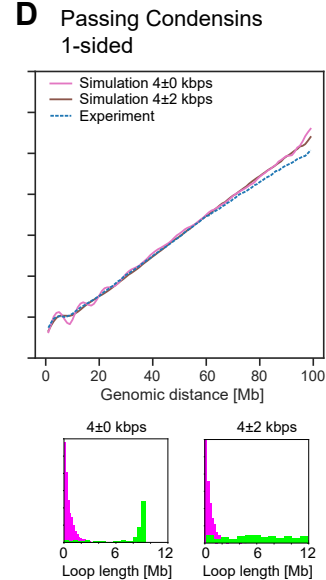**E**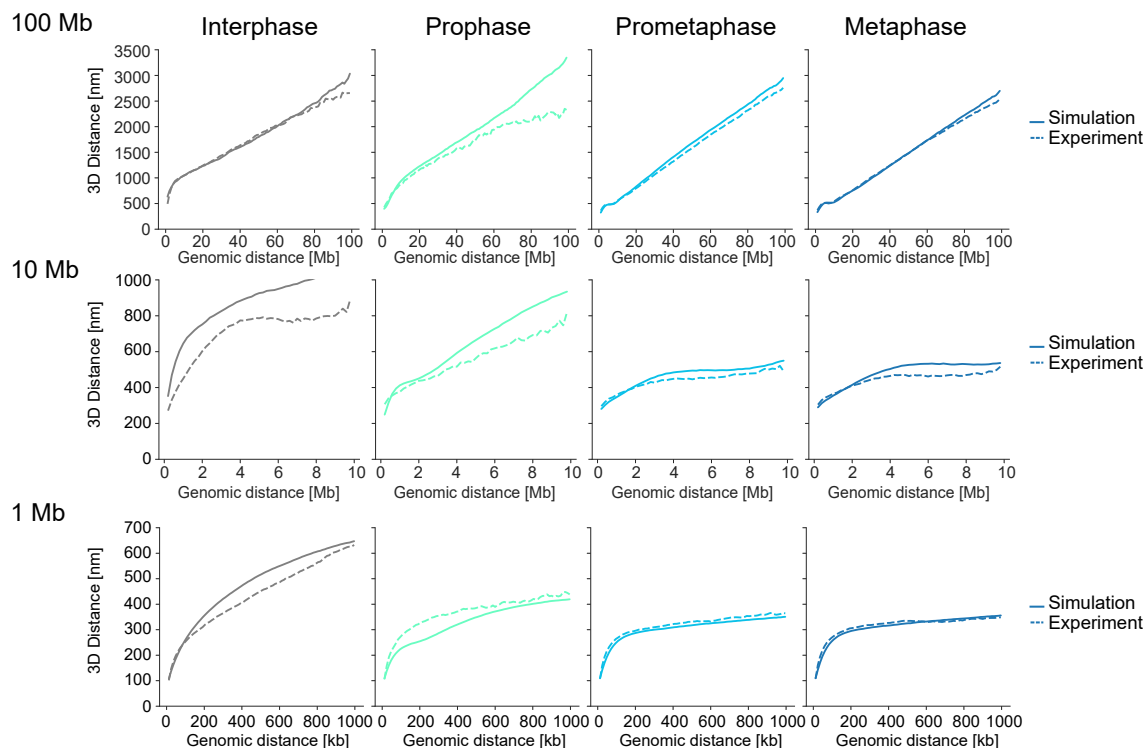**F**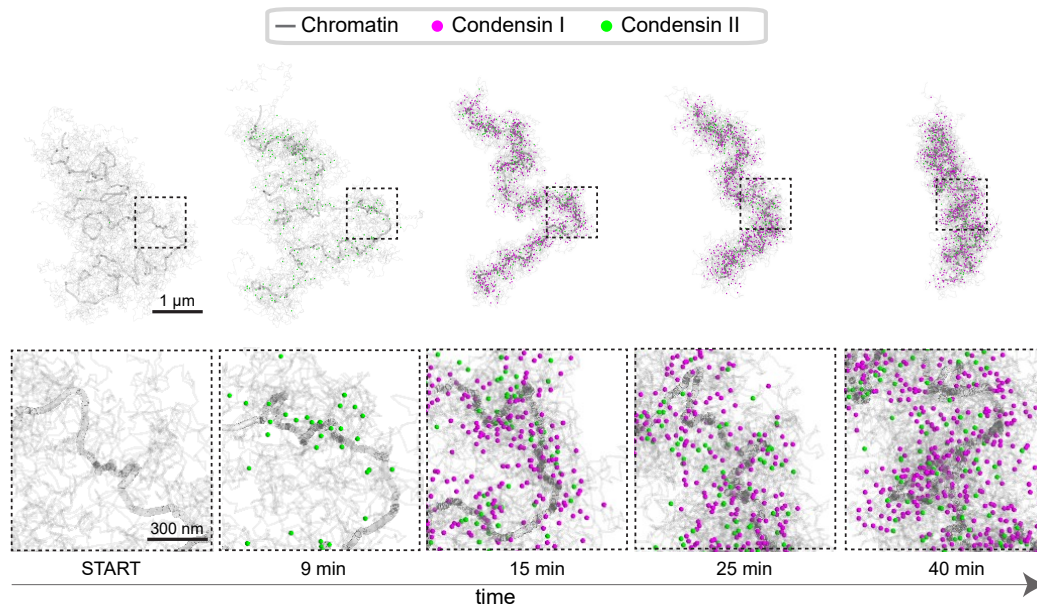**G**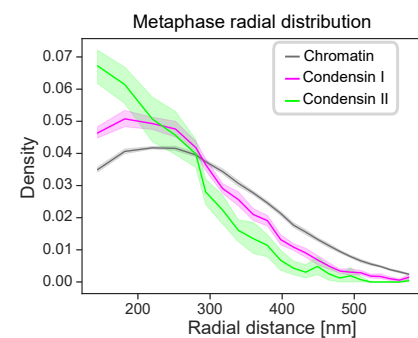

Supplement: 5 — Figure S5. A data-constrained dynamic polymer model of condensin-driven loop extrusion predicts mitotic chromosome organization, related to Figure 5 (A) Distance scaling plot of an unconstrained polymer to experimental DSMC4 metaphase high-resolution tracing data (12-kb resolution, chr5) gave 12 nm/kb monomer and 1 kbT repulsive potential as optimal polymer simulation parameters. Experimental data from 212 (608) DSMC4 cells (traces), 2 independent experiments. (B–D) Distance scaling plots and histograms of condensin I (magenta) and condensin II (green) loop lengths resulting from alternative loop extrusion models, including (B) altered extrusion speed assuming one-sided extrusion and no stalling, (C) one- and two-sided extrusion at 4 ± 2 kbps with full stalling, and (D) altered extrusion speed distributions assuming one-sided extrusion and no stalling. Simulation positions were sampled as experimental data (100-kb spots, 1-Mb resolution) 10 times per 100-Mb chromosome with offset genomic positions. Simulated data from 10 to 20 dynamically simulated metaphase (40 min) chromosomes per condition. Experimental data from chr2q, 100 Mb: n =2 12 (686) cell (traces), 3 independent experiments. (E) Distance scaling plots of simulated chromosomes, assuming one-sided extrusion at 4 ± 2 kbps and no stalling, and corresponding experimental data. Simulated traces were spatially sampled as experimental data (12-kb tiled probes, 30-kb probes with 200-kb resolution, and 100-kb probes with 1-Mb resolution). Sampling time points were 0, 10, 32, and 40 min for interphase, prophase, prometaphase, and metaphase, respectively. Simulated data from n = 20 dynamically simulated 100-Mb chromosomes. Experimental data from HeLa WT cells; see Figures 3 and S3 for detailed information. (F) Ground-truth positions of condensin I (magenta) and condensin II (green) in simulated chromatin (2-kb sampling in light gray, 1-Mb rolling average in dark gray), corresponding to the example in Figure 5A. (G) R [file NIHMS2067674-supplement-5.pdf]

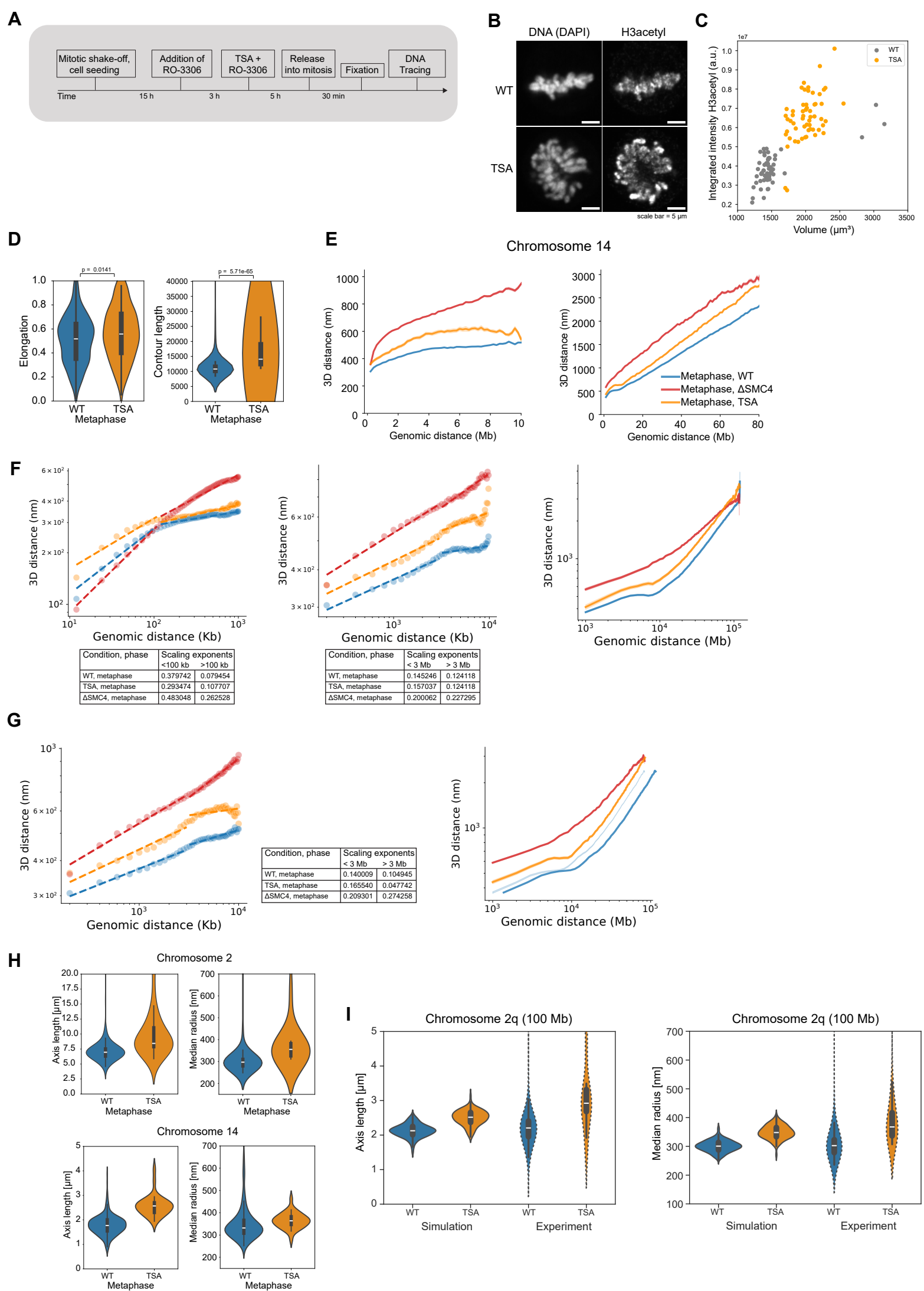

Supplement: 6 — Figure S6. Histone hyperacetylation leads to mitotic chromosome decompaction while preserving condensin-driven features, related to Figure 6 (A) Experiment scheme for the generation of hyperacetylated mitotic chromosomes using TSA treatment during interphase. (B) Fluorescent micrographs of HK WT metaphase cells in WT condition or after 5 h of pre-mitotic TSA treatment. Cells were stained with an anti-histone 3 acetyl antibody to visualize histone acetylation levels. Scale bar, 5 μm. (C) Scatterplot shows a positive correlation between histone acetylation levels and chromosome volume. nWT = 59, nΔSMC4 = 58. (D) Trace metric from chr5:149,500,723–150,699,962 (1.2 Mb, 12-kb resolution) for WT and TSA-treated cells. Data from 152 (398) WT cells (traces), 3 independent experiments, and 67 (168) TSA-treated cells (traces), 2 independent experiments. Median, quartiles, and whiskers are shown in the plots. (E) Distance scaling plots from chr14, 10 Mb and whole-chromosome scales for WT, DSMC4, and TSA-treated cells at metaphase. Chr14, 10 Mb: 69 (133) TSA-treated cells (traces), 2 independent experiments; chr14, whole: 15 (30) TSA-treated cells (traces), one experiment. See Figure S4 for details on WT and DSMC4 data. As the scaling data become very sparse at maximal genomic trace distance, the scaling plots were cropped to 10 and 80 Mb, respectively. (F) Distance scaling plots on log-log scale from chr5 (1-Mb scale), chr2 (10-Mb scale), and chr2q (100-Mb scale), corresponding to Figure 6D. For the 1- and 10-Mb scales, power law fits below and above 100 kb and 3 Mb, respectively, are shown with dashed lines, and fit exponents are shown in the table below the graph. (G) As in (F), with data from chr14 (10-Mb scale) and chr14q (100-Mb scale), corresponding to the data shown in (E). (H) Length and width of WT and TSA-treated metaphase chr2 and chr14, estimated by length of a 20-Mb rolling average axis and median distance of each point to the closest rolling average point from ch [file NIHMS2067674-supplement-6.pdf]
