# Supplementary material for: Nanoscale DNA tracing reveals the self-organization mechanism of mitotic chromosomes
Source: Cell. Author manuscript; Available in PMC 2025 Jun 2. (PMC12127698; doi:10.1016/j.cell.2025.02.028)

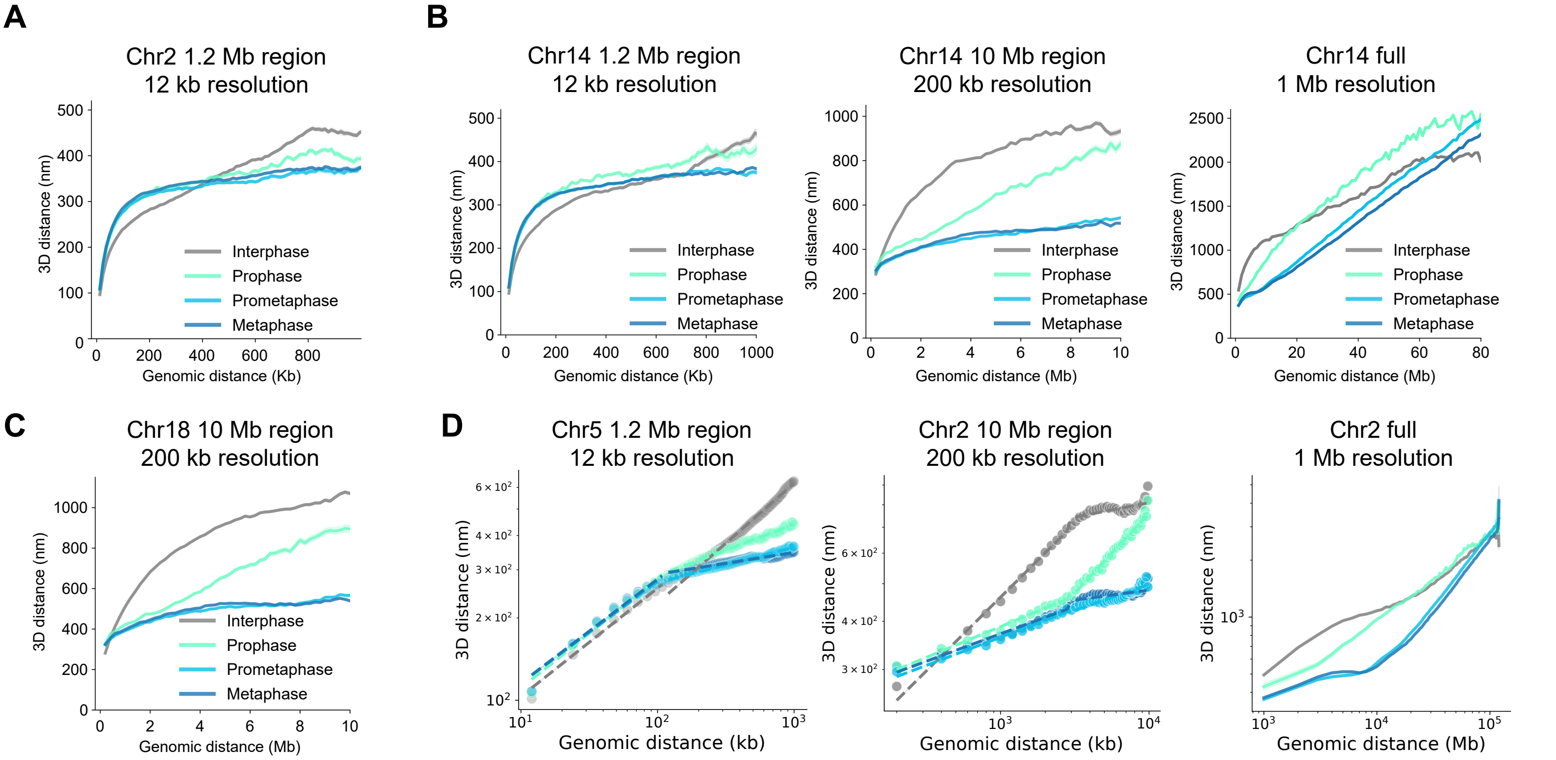

WT, Phase	Scaling exponents <100 kb	>100 kb
Interphase	0.396203	0.438087
Prophase	0.401306	0.188317
Prometaphase	0.371307	0.128748
Metaphase	0.379742	0.079454

WT, phase	Scaling exponents <100 kb	>100 kb
Interphase	0.381633	0.074717
Prophase	0.159090	0.413014
Prometaphase	0.145967	0.134329
Metaphase	0.141941	0.051638

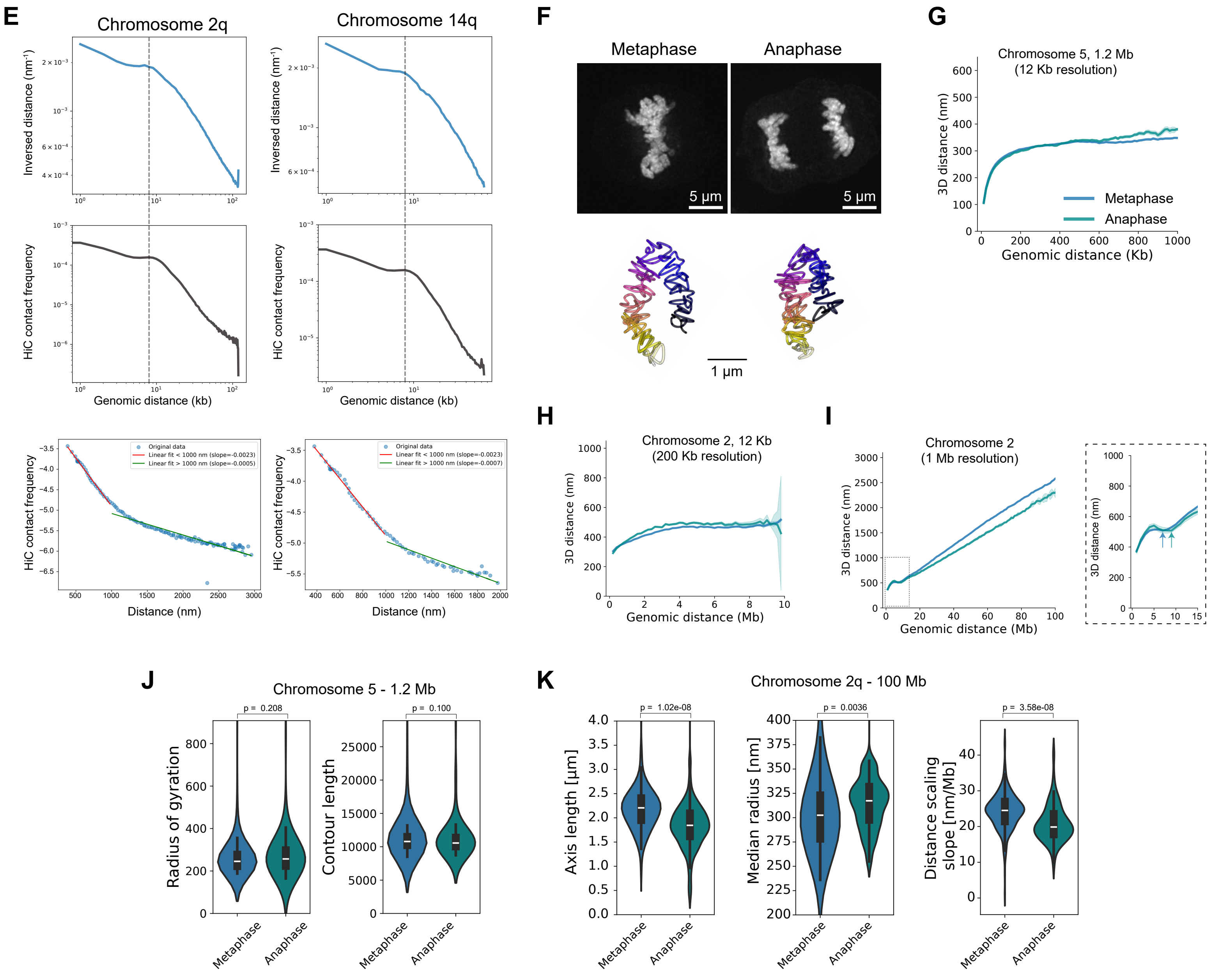

Supplement: 3 — Figure S3. Multiscale chromatin traces reveal mitosis-specific genome scaling behavior, related to Figure 3 (A) Pairwise distance scaling plots for chr2:191,110,000–192,309,940 (12-kb resolution). Data from n = 711 (1,280) cells (traces), 2 independent experiments. Plots show median ± standard error of the mean. (B) Pairwise distance scaling plots for chr14:50,923,646–52,104,342 (12-kb resolution), chr14:45,200,003–56,429,971 (200-kb resolution), and chr14:20,000,036–105,029,664 (1-Mb resolution). Data from n = 677 (1,243) cells (traces), 2 independent experiments; n = 645 (1,201) cells (traces), 2 independent experiments; and n = 543 (1,039) cells (traces), 2 independent experiments, respectively. Plots show median ± standard error of the mean. As the scaling data become very sparse at maximal genomic trace distance, the scaling plots were cropped to 1, 10, and 80 Mb, respectively. (C) Pairwise distance scaling plots for chr18:50,000,077–62,829,816 (200-kb resolution). Data from n = 633 (1,134) cells (traces), 2 independent experiments. Plots show median ± standard error of the mean. (D) Distance scaling plots on log-log scale from chr5 (1-Mb scale), chr2 (10-Mb scale), and chr2q (100-Mb scale), corresponding to Figure 3. For the 1- and 10-Mb scales, power law fits below and above 100 kb and 3 Mb, respectively, are shown with dashed lines, and fit exponents are shown in the table below the graph. No fit was performed on full chromosome data. (E) Top row: inverted median distance scaling for chr2q (left column) and chr14q (right column) in metaphase HeLa cells sampled at 1-Mb resolution. Middle row: HiC P(s) scaling plots from chr2q and chr14q from prometaphase-arrested HeLa cells (Gibcus et al.,11 GEO: GSM2745897), downsampled by averaging to 1-Mb resolution. The dashed line is placed at 8 Mb and indicates the approximate position of the scaling dip (visualized as a bump in this representation). Bottom row: scatterplot of log-transformed HiC frequencies against pai [file NIHMS2067674-supplement-3.pdf]
