# Supplementary material for: Nanoscale DNA tracing reveals the self-organization mechanism of mitotic chromosomes
Source: Cell. Author manuscript; Available in PMC 2025 Jun 2. (PMC12127698; doi:10.1016/j.cell.2025.02.028)
Supplement: 7 [file NIHMS2067674-supplement-7.pdf]

Table S1: Experimental dataset overview, related to Figure 1.

Cell type	Phase	Region	N exp	N cells	N traces	Median trace completenes	Resolution	Genomic size
HK_WT	inter	Chr5:149506000-150694000	3	378	858	0.84	12 kb	1.2 Mb
HK_WT	inter	Chr2:191115000-192303000	2	262	434	0.85	12 kb	1.2 Mb
HK_WT	inter	Chr14:50930000-52102000	2	240	388	0.797979798	12 kb	1.2 Mb
HK_WT	inter	Chr2:185011000-194816000	2	202	334	0.86	200 kb	12 Mb
HK_WT	inter	Chr14:45215000-56418000	2	255	548	0.663461538	200 kb	11 Mb
HK_WT	inter	Chr18:50014000-62815000	2	235	426	0.738461538	200 kb	13 Mb
HK_WT	inter	Chr2:58000-240053000	3	356	1138	0.213389121	1 Mb	240 Mb
HK_WT	inter	Chr14:20015000-105016000	2	237	525	0.333333333	1 Mb	90 Mb
HK_WT	pro	Chr5:149506000-150694000	3	40	116	0.74	12 kb	1 MB
HK_WT	pro	Chr2:191115000-192303000	2	34	69	0.76	12 kb	1 MB
HK_WT	pro	Chr14:50930000-52102000	2	35	74	0.651515152	12 kb	1 MB
HK_WT	pro	Chr2:185011000-194816000	2	32	60	0.82	200 kb	12 Mb
HK_WT	pro	Chr14:45215000-56418000	2	35	81	0.846153846	200 kb	11 Mb
HK_WT	pro	Chr18:50014000-62815000	2	36	65	0.738461538	200 kb	13 Mb
HK_WT	pro	Chr2:58000-240053000	3	29	102	0.282426778	1 Mb	240 Mb
HK_WT	pro	Chr14:20015000-105016000	2	23	51	0.452380952	1 Mb	90 Mb
HK_WT	prometa	Chr5:149506000-150694000	3	227	628	0.78	12 kb	1 MB
HK_WT	prometa	Chr2:191115000-192303000	2	182	382	0.75	12 kb	1 MB
HK_WT	prometa	Chr14:50930000-52102000	2	180	387	0.717171717	12 kb	1 MB
HK_WT	prometa	Chr2:185011000-194816000	2	174	368	0.9	200 kb	12 Mb
HK_WT	prometa	Chr14:45215000-56418000	2	186	425	0.884615385	200 kb	11 Mb
HK_WT	prometa	Chr18:50014000-62815000	2	180	406	0.761538462	200 kb	13 Mb
HK_WT	prometa	Chr2:58000-240053000	3	238	903	0.284518828	1 Mb	240 Mb
HK_WT	prometa	Chr14:20015000-105016000	2	186	501	0.535714286	1 Mb	90 Mb
HK_WT	meta	Chr5:149506000-150694000	3	227	664	0.87	12 kb	1 MB
HK_WT	meta	Chr2:191115000-192303000	2	211	451	0.86	12 kb	1 MB
HK_WT	meta	Chr14:50930000-52102000	2	207	441	0.787878788	12 kb	1 MB
HK_WT	meta	Chr2:185011000-194816000	2	198	406	0.94	200 kb	12 Mb
HK_WT	meta	Chr14:45215000-56418000	2	213	476	0.913461538	200 kb	11 Mb

HK_WT	meta	Chr18:50014000-62815000	2	204	436	0.815384615	200 kb	13 Mb
HK_WT	meta	Chr2:58000-240053000	3	208	820	0.292887029	1 Mb	240 Mb
HK_WT	meta	Chr14:20015000-105016000	2	195	492	0.553571429	1 Mb	90 Mb
HK_WT	ana	Chr5:149506000-150694000	3	61	128	0.885	12 kb	1 MB
HK_WT	ana	Chr2:191115000-192303000	2	49	85	0.89	12 kb	1 MB
HK_WT	ana	Chr14:50930000-52102000	2	44	71	0.838383838	12 kb	1 MB
HK_WT	ana	Chr2:185011000-194816000	2	41	75	0.96	200 kb	12 Mb
HK_WT	ana	Chr14:45215000-56418000	2	51	94	0.942307692	200 kb	11 Mb
HK_WT	ana	Chr18:50014000-62815000	2	43	76	0.892307692	200 kb	13 Mb
HK_WT	ana	Chr2:58000-240053000	3	74	214	0.276150628	1 Mb	240 Mb
HK_WT	ana	Chr14:20015000-105016000	2	57	109	0.44047619	1 Mb	90 Mb
HK_dSMC4	meta	Chr5:149506000-150694000	2	181	615	0.78	12 kb	1 MB
HK_dSMC4	meta	Chr2:185011000-194816000	2	113	199	0.92	200 kb	12 Mb
HK_dSMC4	meta	Chr14:45215000-56418000	2	185	498	0.903846154	200 kb	11 Mb
HK_dSMC4	meta	Chr2:58000-240053000	2	174	616	0.274058577	1 Mb	240 Mb
HK_dSMC4	meta	Chr14:20015000-105016000	1	81	176	0.595238095	1 Mb	90 Mb
HK_TSA	meta	Chr5:149506000-150694000	2	68	203	0.87	12 kb	1 MB
HK_TSA	meta	Chr2:185011000-194816000	2	69	140	0.92	200 kb	12 Mb
HK_TSA	meta	Chr14:45215000-56418000	2	69	143	0.884615385	200 kb	11 Mb
HK_TSA	meta	Chr2:58000-240053000	2	24	93	0.326359833	1 Mb	240 Mb
HK_TSA	meta	Chr14:20015000-105016000	1	15	34	0.81547619	1 Mb	90 Mb

Table S2: Simulation parameters, related to Figure 5.

Loop extrusion simulation parameters

Parameter	Value	Reference
Condensin-I abundance	9.6/Mb	[9,22]
Condensin-II abundance	2.4/Mb	[9,22]
Condensin-I residence time (prophase)	0 s	[9]
Condensin-I residence time (prometa-/metaphase)	150 s	[9]
Condensin-II residence time (pro-/prometa-/metaphase)	3600 s	[9]
Extrusion rate	1-4 ± 0-2 kbps, see main text	[32]
(A)symmetric extrusion	One- or two-sided, see main text	[8,32]
Stall probability	0-100%, see main text	[32]
Duration prophase	10 min	[9,31]
Duration prometa- and metaphase	30 min	[9,31]
Simulation time-step	1 s	This work

Polymer simulation parameters

Parameter	Value	Reference
Number of monomers	100 000 (defined to 1kb/monomer)	This work
Chain/loop bond type	Harmonic bond	Polychrom documentation
Chain bond distance	1 (scaled to 12 nm in analysis)	This work
Chain bond standard deviation	10 % of chain bond	Polychrom documentation
Loop bond distance	50 % of chain bond	Polychrom documentation
Loop bond standard deviation	20 % of chain bond	Polychrom documentation
Periodic boundary conditions	Not used	This work
Collision rate	0.03	Polychrom documentation
Error tolerance	0.01	Polychrom documentation
Angle force	1.5	Polychrom documentation
Repulsive force type	Polynomial repulsive	Polychrom documentation
Repulsive force WT	1	Polychrom documentation
Repulsive radius WT	1.05	Polychrom documentation
Repulsive force TSA	10	This work
Repulsive radius TSA	1.75	This work
Initial polymer equilibration steps	100 000 steps	This work
MD steps per loop extrusion step	20 000 steps	This work
Sampling frequency of loop extrusion steps	Every 5 seconds	This work
